# T Cell Immune Profiles of Blood and Tumor in Dogs Diagnosed With Malignant Melanoma

**DOI:** 10.3389/fvets.2021.772932

**Published:** 2021-12-02

**Authors:** Ellen E. Sparger, Hong Chang, Ning Chin, Robert B. Rebhun, Sita S. Withers, Hung Kieu, Robert J. Canter, Arta M. Monjazeb, Michael S. Kent

**Affiliations:** ^1^Department of Medicine and Epidemiology, School of Veterinary Medicine, University of California, Davis, Davis, CA, United States; ^2^Center for Companion Animal Health, Department of Surgical and Radiological Sciences, School of Veterinary Medicine, University of California, Davis, Davis, CA, United States; ^3^California National Primate Research Center, Department of Medical Microbiology and Immunology, University of California, Davis, Davis, CA, United States; ^4^Department of Veterinary Clinical Sciences, School of Veterinary Medicine, Louisiana State University, Baton Rouge, LA, United States; ^5^Surgical Oncology, School of Medicine, University of California, Davis, Sacramento, CA, United States; ^6^Radiation Oncology, School of Medicine, University of California, Davis, Sacramento, CA, United States

**Keywords:** melanoma, canine, dog, immune profile, one health, comparative oncology

## Abstract

Investigation of canine T cell immunophenotypes in canine melanomas as prognostic biomarkers for disease progression or predictive biomarkers for targeted immunotherapeutics remains in preliminary stages. We aimed to examine T cell phenotypes and function in peripheral blood mononuclear cells (PBMC) and baseline tumor samples by flow cytometry, and to compare patient (*n* = 11–20) T cell phenotypes with healthy controls dogs (*n* = 10–20). CD3, CD4, CD8, CD25, FoxP3, Ki67, granzyme B, and interferon-γ (IFN-γ) were used to classify T cell subsets in resting and mitogen stimulated PBMCs. In a separate patient cohort (*n* = 11), T cells were classified using CD3, CD4, CD8, FoxP3, and granzyme B in paired PBMC and single cell suspensions of tumor samples. Analysis of flow cytometric data of individual T cell phenotypes in PBMC revealed specific T cell phenotypes including FoxP3+ and CD25+FoxP3- populations that distinguished patients from healthy controls. Frequencies of IFN-γ+ cells after ConA stimulation identified two different patient phenotypic responses, including a normal/exaggerated IFN-γ response and a lower response suggesting dysfunction. Principle component analysis of selected T cell immunophenotypes also distinguished patients and controls for T cell phenotype and revealed a clustering of patients based on metastasis detected at diagnosis. Findings supported the overall hypothesis that canine melanoma patients display a T cell immunophenotype profile that is unique from healthy pet dogs and will guide future studies designed with larger patient cohorts necessary to further characterize prognostic T cell immunophenotypes.

## Introduction

Immunotherapy has shown great promise in the treatment of melanoma in humans, including those with an advanced stage of disease, but a majority of patients still fail to respond long term to these therapies and a high rate of toxicity is associated with their use (1–4). Elucidation of the most critical immunologic components of the immune environment that impact response to such therapeutics will be essential for increasing response rates and duration of responses (5). The canine cancer model affords a unique opportunity to characterize immunologic phenotypes and biomarkers that may be predictive of responses to specific immunotherapeutics, particularly for malignant melanoma. Spontaneously arising melanoma in the dog has been shown to be a viable model system for human melanoma, with canine oral melanoma and human mucosal melanoma being the most studied (6–10). Oral melanoma is the most commonly occurring malignant tumor in the oral cavity of dogs (11). The majority of these canine tumors are malignant in nature with a high metastatic rate, although a more benign variant has been reported (12). Despite standard chemotherapy, radiotherapy and clinical trials with traditional cancer vaccines (13–16), the majority of dogs with oral melanoma die of their disease as was the case in human medicine prior to the advent of more targeted immunotherapies, although most patients in human medicine still are not cured despite these advances (4, 17).

Knowledge of dog immunology and tumor immunology in particular, has been limited by the availability of reagents for canine immune markers and validated flow cytometry panels (18). Reagents for identification of canine lymphocyte subsets have evolved from veterinary investigations focused on characterizing and typing of canine and feline lymphomas (19–24) over the past 30 years. However, additional canine-specific or cross-reactive reagents for critical immune markers are now emerging rapidly (25–32). These new reagents and tools have encouraged the development of diagnostic and experimental tools (33–37). This will facilitate examination of the canine immune response to melanoma and immunologic interventions for multiple cancers including melanoma. In turn, these reagents support and encourage the use of this model to investigate novel immunologic interventions that will impact naturally occurring cancers in pet dogs as well as human cancer.

It is well-understood that host T cell immunity plays a critical role in control and progression of cancer in humans (38–40). The emergence of cancer immunotherapeutics that target cancer-related T cell dysregulation such as the currently approved immune checkpoint inhibitors based on PD1, PD-L1, and CTLA-4 have encouraged the investigation of additional checkpoint pathways as immunotherapeutic targets. Naturally occurring canine oral melanoma may provide a very useful model for testing of emerging or very novel therapeutics (41, 42). Furthermore, these studies may provide viable alternative therapeutics for certain intractable canine cancers such as oral melanoma. However, investigations of canine T cell subsets and their role as biomarkers for canine melanoma progression or response to immunotherapeutics, remain in early stages. The aims of this preliminary study were to examine T cell phenotypes and function in peripheral blood mononuclear cells (PBMC) and tumor tissue in untreated cases of melanoma by use of flow cytometry panels, and to compare T cell phenotypes of canine melanoma patients with healthy control dogs. The study was designed to test the overall hypothesis that canine melanoma patients demonstrate a T cell immunophenotype that is different and unique from healthy pet dogs. Our studies revealed specific T cell phenotypes that do distinguish canine melanoma patients from healthy dogs.

## Materials and Methods

### Canine Melanoma Patient and Healthy Control Cohorts

Dogs presenting to the University of California Davis (UC Davis) William R. Pritchard Veterinary Medical Teaching Hospital (VMTH) with a histological diagnosis of a malignant melanoma and gross disease were recruited to the study. Dogs were required to have no prior treatment of their melanoma. Blood and tissue were collected with informed owner consent under approval from the UC Davis Institutional Animal Care and Use Committee (IACUC) and Clinical Trials Review Board (protocols 22172 and 22002). Healthy client owned dogs served as controls and were recruited through clients and staff of the UC Davis VMTH and blood was drawn with informed consent and IACUC approval (22172). Demographic and tumor specific information for patient and control dogs is presented in Supplementary Table 1.

### Isolation of Canine Peripheral Blood Mononuclear Cells (PBMC) From Whole Blood

Whole blood was obtained from canine melanoma patients and healthy dogs using vacutainer tubes containing EDTA anticoagulant. Blood was processed as described previously (25). Briefly, PBMC were isolated by density centrifugation using Histopaque 1077 (Sigma-Aldrich, Saint Louis, MO, USA). PBMC pellets were further processed by treatment with RBC lysis buffer (Biolegend, San Diego, CA, USA). Isolated PBMC were subsequently tested as fresh PBMC by flow cytometric analysis, or cryopreserved in media containing 45% heat inactivated fetal bovine serum (FBS), 45% heat inactivated dog serum (Equitech-bio, Kerrville, TX, USA), and 10% DMSO. Cryopreserved PBMC were used for all studies with the exception of a single study comparing tumor associated cells and PBMC from a select patient cohort where fresh tumor associated cells and fresh PBMC from patients (and controls) were stained and tested. Fresh PBMC or thawed cryopreserved PBMC were rested/cultured in culture media overnight prior to staining the next day for flow cytometry analysis as described previously (33).

### Preparation of Tumor Samples for Flow Cytometry Analysis

Canine melanoma tumor biopsy samples were cut into small pieces in Dulbecco's phosphate-buffered saline (DPBS) (Gibco, Carlsbad, CA, USA) and digested in DPBS containing 1 mg/ml collagenase IV, 0.5% bovine serum albumin, with or without 0.1 mg/ml DNase for 2 h on a shaker at 37°C. Tumor associated cells were washed with DPBS, filtered by a 70 μm filter, and tumor cells were stained on the same day.

### Stimulation of PBMC With Concanavalin A (ConA)

PBMC were treated with ConA (Sigma-Aldrich) (5 μg/ml) and brefeldin A (Sigma-Aldrich) (1 μg/ml) in cell culture media for 16 h at 37°C before assay for expression of interferon (IFN-γ) by intracellular cytokine staining (ICS) the next day. Unstimulated control PBMC for each sample (patient and control) were incubated in media with brefeldin A and without ConA for 16 h at 37°C.

### Flow Cytometry

Staining protocols were previously described (25, 33). Briefly, when possible a minimum of 1 million cells were stained for PBMC and 2 million cells were stained for tumor associated cells. For all experiments, cells were stained for viability with a fixable viability dye (LIVE/DEAD™ Fixable Aqua Dead Cell Stain Kit, or LIVE/DEAD™ Fixable Near-IR Dead Cell Stain Kit, Thermo Fisher Scientific, Waltham, MA, USA). Cells were stained with cell surface antibodies in DPBS with 3% heat inactivated fetal bovine serum (FBS). Permeabilization, fixation and intracellular staining of cells were performed using eBioscience™ Foxp3 / Transcription Factor Staining Buffer Set (Thermo Fisher Scientific). Cell surface staining, permeabilization, fixation, and intracellular staining were performed for 20 min at 4°C.

Antibodies staining for cell surface markers were directed against CD4, CD8α, and CD25. Antibodies staining for intracellular markers were directed against CD3, FoxP3, Ki67, granzyme B, and IFN-γ. One panel included a fixable viability dye (Aqua or Near-IR) and antibodies for CD3, CD4, CD8, CD25, FoxP3, and Ki67. A second panel contained the same markers and in addition, antibodies for detection of granzyme B and IFN-γ. These two panels were utilized for PBMC. Tumor associated cells and corresponding PBMC were tested with a fixable viability dye and antibodies for CD3, CD4, CD8, FoxP3, and granzyme B. All antibodies are described for species, clone number, fluorochrome, and vendor in Table 1. Of note, PBMC from four melanoma patients were tested only with the anti-human CD25 antibody.

**Table 1:** Antibodies used in flowcytometry panels.

**Target/species**	**Conjugates**	**Clone #**	**Vendor**
CD3/Human	FITC	CD3-12	Bio-Rad
CD4/Dog	Pacific Blue	YKIX302.9	Bio-Rad
CD4/Dog	PE-Cy7	YKIX302.9	Bio-Rad
CD8a/Dog	Alexa Fluor 700	YCATE55.9	Bio-Rad
CD8a/Dog	PerCP-eFluor 710	YCATE55.9	Thermo Fisher
CD25/Human	PE	ACT1	Dako
CD25/Dog	PE	P4A10	Thermo Fisher
Foxp3/Human	Alexa Fluor 700	FJK-16s	Thermo Fisher
Foxp3/Human	eFluor 660	FJK-16s	Thermo Fisher
Granzyme B/Human	PE-Texas Red	GB11	Thermo Fisher
Interferon-γ /Bovine	PE	CC302	Bio-Rad
Ki67/Human	PE-Cy7	20Raj1	Thermo Fisher

Stained PBMC were washed in buffers and under conditions as previously described (33), suspended in 1% paraformaldehyde (Affymetrix, Thermo Fisher Scientific), and stored at 4°C for subsequent flow cytometer acquisition. A BD LSRII flow cytometer (BD Biosciences, San Jose, CA, USA) was utilized for most flow acquisitions with a BD Fortessa flow cytometer (BD Biosciences) serving as an alternative when the BD LSRII was unavailable for use. For PBMC, minimum acquisition events were 63,000. For tumor associated cells, minimum acquisition events were 26,000. Typically, 100,000 events were acquired. Acquired flow cytometric data utilized FlowJo v10.6.1 (BD Biosciences) software.

### Statistics

Data was collected in a commercially available spreadsheet software program (Microsoft Excel, Microsoft Corporation, Redmond, WA, USA) and descriptive statistics were calculated. Initial exploration of the data was done using a principal component analysis (PCA). PCA was performed using R version 4.1.0 (R Core Team (2021). R: A language and environment for statistical computing. R Foundation for Statistical Computing, Vienna, Austria. https://www.R-project.org/). PCA plots were generated using R package ggplot2_3.3.3 (43). PERMANOVA was performed using the default parameters with the adonis function and beta-dispersion was calculated using the permutest function with the betadisper function in the vegan_2.5-7 package (vegan: Community Ecology Package. R package version 2.5-7. https://CRAN.R-project.org/package=vegan). Data was checked for normality using a Shapiro-Wilks test. Non-parametric analysis was performed as most data sets did not meet the criteria of normality. To determine differences between patients and controls, a pair-wise analysis was conducted using the Mann Whitney *U*-test. To investigate differences in flow cytometry parameters between matched tumor and PBMC, a Wilcoxon matched-pairs signed rank test was used. Comparisons of frequencies between three or more subsets were performed using a Kruskal-Wallis test with the Dunn's *post-hoc* test for multiple comparisons. To test for differences in the demographic data between continuous variables between patients and controls, a Mann Whitney *U*-test was performed while a Fischer's exact test was done to look for differences in proportions of categorical data between patients and controls. Statistical tests and graphing utilized GraphPad Prism software (GraphPad Prism version 9.2, GraphPad Software, San Diego, CA USA) for flow cytometry data and Stata (Stata/IC version 14.2., StataCorp, College Station, TX, USA) for demographic and clinical tumor related data. A *p*-value < 0.05 was considered statistically significant.

## Results

### Cohort Description

A total of 31 dog patients diagnosed by histopathology with a malignant melanoma and 23 healthy control dogs were enrolled. Healthy dogs were followed for at least 1 year after sample collection to ensure that they did not develop a melanoma. The median age of patients at sample collection was 10.2 years (range 5.9–15.1 years) while the median age of control dogs at time of sample collection was 8.5 years (range 4–15.5 years). These were statistically different at *p* = 0.03. Amongst the patients there was 1 intact female dog, 14 female spayed dogs, 15 castrated male dogs and one intact male, while for the control group there was 11 female spayed dogs and 12 castrated male dogs. The proportions of dogs in each category were not different at *p* = 1.0. In the patient group there were 10 mixed breed dogs, four Labrador retrievers, and a range of other breeds (Supplementary Table 1). In the control group there were seven mixed breed dogs, three rat terriers, three golden retrievers, and one or two of other breeds as listed in Supplementary Table 1. Weights were available for 31 of the cases and 19 of the control dogs. The median weight in the patient group was 24.7 kg (range 4.9–50 kgs) while the median weight in the control group was 14.9 kg (range 5.2–47 kg). These were not statistically different at *p* = 0.24.

Twenty-six patients were diagnosed with an oral melanoma, two had dermal melanomas, and one each of a digital and anal gland melanoma and in one dog the primary could not be identified with a diagnosis made by biopsy of an enlarged submandibular lymph node. Twenty-nine of the cases had primary tumor measurements available. The median largest diameter tumor was 2.8 cm (range 0.5–7 cm). Mitotic index was available for 22 cases. The median number of mitotic figures in 10 high-power fields was 6.5 (range 0–40). Ten cases had metastasis at the time of diagnosis.

### Development of Staining Panels for Flow Cytometric Analysis of Canine T Cells in Blood

**Figure 1:**
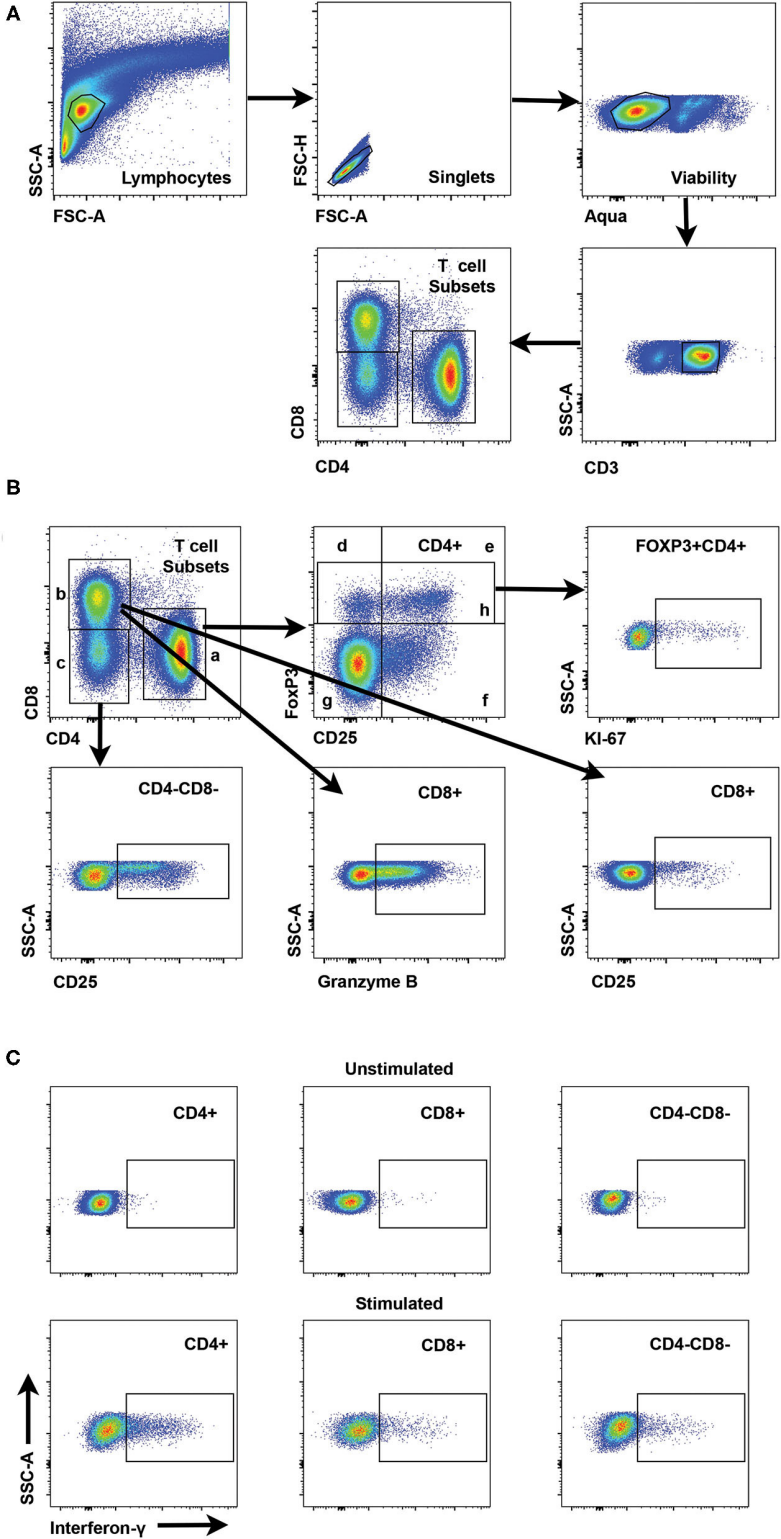
Gating strategies for interrogation of canine T cell subsets by flow cytometry. **(A)** Representative scatter plots reveal the gating strategy for detection of CD4+, CD8+, and CD4-CD8- T cell subsets starting with establishment of a lymphocyte gate, followed by gating on singlets, exclusion of dead cells by a viability stain and gating on CD3+ cells as the parental gate for T cell subsets. **(B)** Scatter plots showing interrogation of CD4+ T cells are representative for gating of specific populations for all T cell subsets. Regulatory CD4+ T cells include FoxP3+ (h) and CD25+FoxP3+ (e). CD25+FoxP3- cells (f) represent an activated T cell subset and CD25-FoxP3- cells (g) are designated as a quiescent T cell subset. Interrogation of FoxP3+ cells for Ki67 reveals the gating strategy for Ki67+ cells for all subsets. Gating strategies for CD25 and Granzyme B are shown for both CD4-CD8- and CD8+ T cells. **(C)** Representative scatter plots show interferon-γ staining and gating for CD4+, CD8+ and CD4-CD8- T cell subsets with or without stimulation with Con-A.

Flow cytometry panels were developed for phenotyping canine T cells in cryopreserved PBMC isolated from blood to explore changes in regulatory and activated T cell populations in canine melanoma patients and healthy dogs. Representative gating strategies are shown for characterizing frequencies of T cell (CD3+) subsets including CD4+, CD8+, and CD4-CD8- cells (Figure 1A) and frequencies of regulatory and activated populations within each T cell subset (Figure 1B). The CD4-CD8- T cell subset was included in analyses for different T cell markers as a population of interest based on a recent report (44) and was consistently observed in all patient and control blood samples. Based on these gating strategies different T cell subsets were interrogated for FoxP3, CD25, Ki67, and granzyme B to distinguish regulatory, activated, and putative quiescent subsets. Regulatory T cell (Treg) populations were identified as FoxP3+ (including both CD25- and CD25+ cells) or CD25+FoxP3+. Activated subsets were based on expression of CD25 in the absence of FoxP3 expression (CD25+FoxP3-) and quiescent populations as CD25-FoxP3-. All T cell subsets and derivative populations were also analyzed for Ki67 expression as a marker for proliferation (45). CD8+ T cells were assessed for granzyme B expression which can be considered both an activation and exhaustion marker for this subset (46–50) (Figure 1B). A subsequent panel included IFN-γ staining using ICS in addition to regulatory and activation markers to explore IFN-γ expression after ConA stimulation as a marker for T cell function and T cell programming (Figure 1C). A preliminary panel included an anti-human CD25 monoclonal antibody (clone ACT1) reported to be cross-reactive for canine T cells (51–53). However, a subsequent analysis comparing CD25 staining by this anti-human CD25 to an anti-canine CD25 monoclonal antibody (clone P4A10) (54) revealed that the anti-human antibody failed to detect all CD25+ CD4+ T cells as shown in Supplementary Figure 1, although some level of cross-reactivity was observed. A representative staining pattern by the anti-human CD25 antibody Supplementary Figure 1A revealed that detection of the CD25+FoxP3- population was particularly less efficient compared to the anti-canine antibody (Supplementary Figure 1B). Overall staining of CD4+ T cells by the anti-human CD25 antibody resulted in significantly lower frequencies compared to staining by the anti-canine antibody (*p* < 0.0001) (Supplementary Figures 1C,D). Use of this anti-human CD25 in the initial patients resulted in loss of data for this marker in a small subset of patients (*n* = 4) (Supplementary Table 2). Supplementary Tables 1, 2 describe patient and control cohorts and which markers were tested for each cohort.

### Principal Component Analysis (PCA) of Canine Melanoma Patients Reveals a Distinctive T Cell Phenotype in Blood Compared to Healthy Dogs

**Figure 2:**
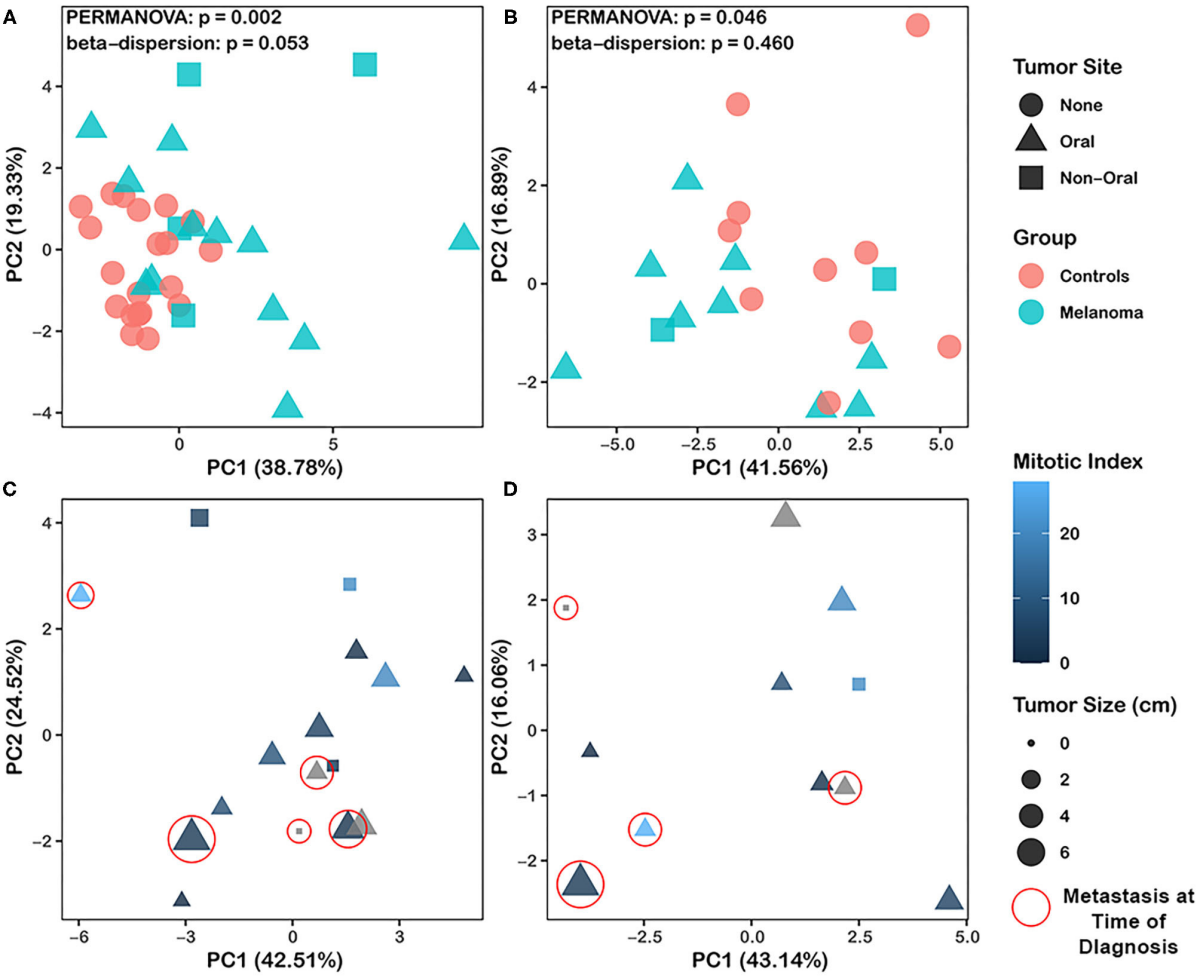
Principal component analysis (PCA) of immunophenotype in melanoma and healthy control patients. **(A)** PCA including 17 immunophenotypes and 36 animals (20 healthy controls, 16 melanoma patients) is shown. **(B)** PCA including 23 background subtracted immunophenotypes after stimulation with ConA and 21 animals (10 healthy controls, 11 melanoma patients) is shown. **(A,B)** Patients are discriminated for melanoma site. Orange circles represent healthy control animals; teal-colored dots denote melanoma patients, which are further categorized into oral melanoma (triangle) and non-oral melanoma (square). **(C)** PCA including 17 immunophenotypes and 16 melanoma patients. **(D)** PCA including 22 background subtracted immunophenotypes after stimulation with ConA and 11 melanoma patients. **(C,D)** Patients are discriminated for melanoma site, tumor mitotic index, tumor size, and metastasis. Triangle points represent patients with oral melanoma; square points represent patients with non-oral melanoma. Points are colored based on mitotic index, from dark to bright blue. Patients with missing mitotic index are denoted by gray color. Size of data points correspond to size of the tumor. Patients with metastasized melanoma are circled in red. PCA was performed using R version 4.1.0. *P*-values < 0.05 are considered significant.

Principal component analysis (PCA) was performed to determine whether canine melanoma patients and healthy controls expressed different T cell immunophenotypes. PCA including 17 immunophenotypes identified as specific T cell subset frequencies (Supplementary Table 3) determined by flow cytometry and 36 animals including 20 healthy controls and 16 melanoma patients which are described in Supplementary Tables 1, 2, revealed a significant difference between healthy controls and canine melanoma patients (PERMANOVA, *p* = 0.002) (Figure 2A). These results suggested that melanoma patients display an overall T cell phenotype in blood that is distinguishable from that of healthy dogs. Furthermore, PCA of 23 immunophenotypes or T cell subset frequencies (Supplementary Table 3) observed after ConA stimulation was conducted for a subset of melanoma patients (*n* = 11) and healthy dogs (*n* = 10) (Supplementary Tables 1, 2), and also revealed a significant difference between healthy controls and melanoma patients (PERMANOVA, *p* = 0.046) (Figure 2B). These findings supported that canine melanoma patients demonstrate a T cell phenotype that is unique and different when compared to healthy dogs.

PCA was also performed on samples from melanoma patients (*n* = 16) with 17 immunophenotypes (same immunophenotypes and patients as described for Figure 2A) to determine if clinical parameters were associated with immunophenotypes in melanoma patients. No significant associations were found between oral vs. non-oral tumor, tumor size, mitotic index or metastasis at time of diagnosis, with immunophenotype (PERMANOVA, *p* > 0.05) (Figure 2C). However, PCA of melanoma patients (*n* = 11) (same patients described for Figure 2B) with 22 immunophenotypes post ConA stimulation (Supplementary Table 3) showed clustering of animals with metastasis at time of diagnosis (*n* = 4) compared to animals without metastasis (*n* = 7) (PERMANOVA, *p* = 0.032) (Figure 2D). Similar to analysis shown in Figure 2C, no significant associations of tumor site, tumor size, or mitotic index with immunophenotype were observed for this PCA (PERMANOVA, *p* > 0.05) (Figure 2D).

### Canine Melanoma Patients Demonstrated Increased Frequencies for Selected Regulatory T Cell Subsets in Blood

**Figure 3:**
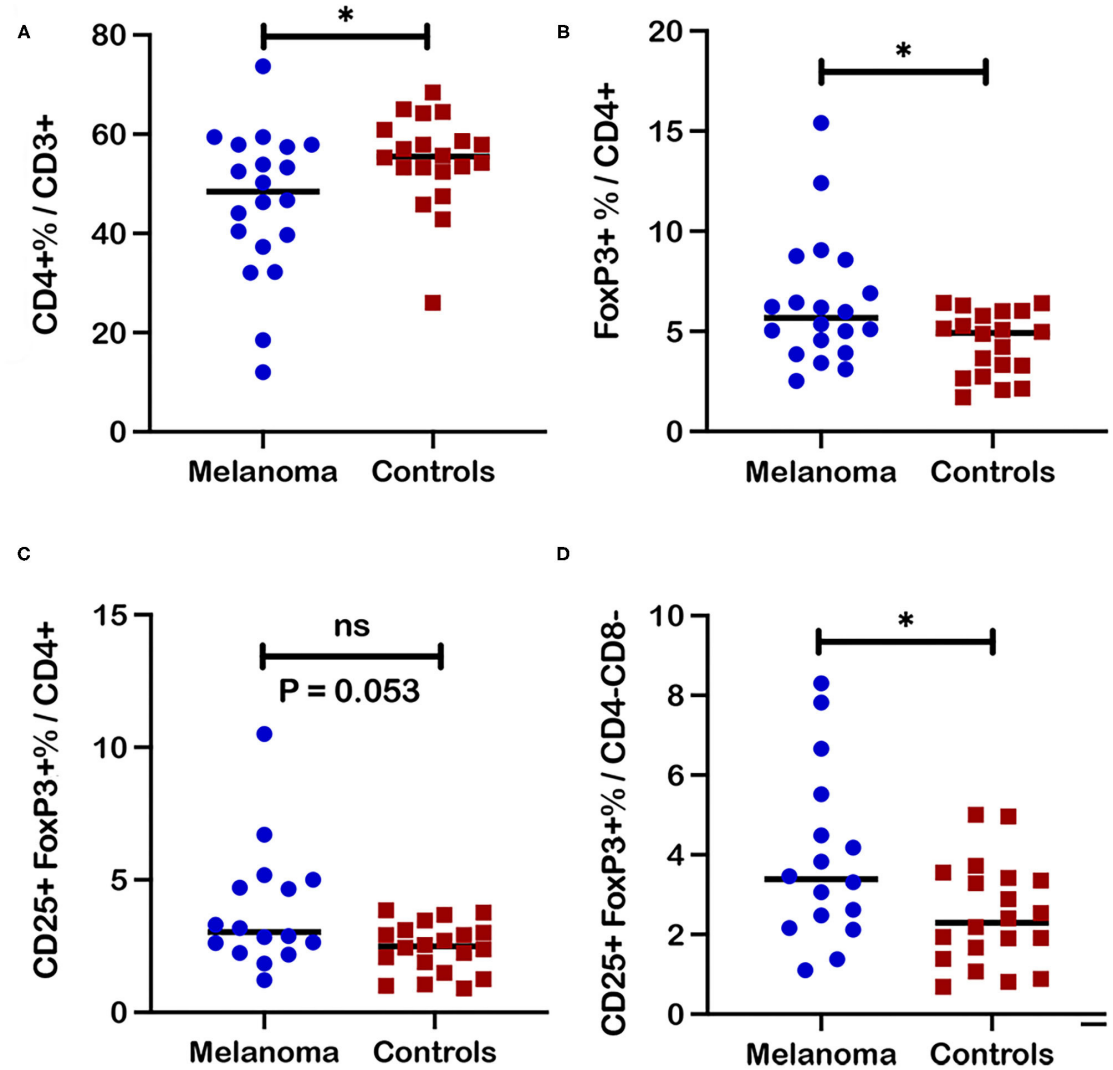
Frequencies of regulatory T cell subsets in blood from canine melanoma patients and healthy controls. **(A)** A comparison of median frequencies for CD4+ cells within the T cell population for patients and controls is shown. **(B)** Median frequencies of FoxP3+ and **(C)** CD25+FoxP3+ cells within the CD4+ T cell population are also compared between patients and healthy controls. **(D)** Median frequencies of CD25+FoxP3+ cells within the CD4-CD8- T cell population are compared between patients and healthy controls. Pair-wise analysis between patients and controls was conducted with the Mann Whitney test using GraphPad Prism software and two-tailed analysis. “ns” denotes not significant. A single asterisk (*) denotes a *P*-value < 0.05. *P*-values < 0.05 are considered significant.

Further assessment of a canine melanoma patient T cell immunophenotype involved comparison analysis of individual T cell subset frequencies between patients and healthy control dogs. Analysis of canine melanoma patients (*n* = 20; P1-20) revealed decreased frequencies of CD4+ T cells compared to healthy controls (*n* = 20, *p* = 0.039) (Figure 3A); however, no significant differences for frequencies of CD8+ and CD4-CD8- T cell subsets in blood were detected between patients and controls (*n* = 20) (Supplementary Figures 2A,B). For examination of Treg subsets, T cell subsets were analyzed with FoxP3 as a single marker (FoxP3+) or for co-expression of CD25 and FoxP3 (CD25+FoxP3+). An increased frequency of FoxP3+ cells within the CD4+ subset was detected for patients compared to controls (*n* = 20, *p* = 0.038) (Figure 3B). Assessment of the regulatory CD25+FoxP3+ population also showed a trend for an increased frequency in the CD4+ subset (*p* = 0.053) (Figure 3C) and an increased frequency for the CD4-CD8- subset (*p* = 0.036) (Figure 3D) for patients (*n* = 16, P5-20) compared to controls (*n* = 20). Significant differences between patients and controls for regulatory T cell subsets were modest and were not detected for FoxP3+ populations within the CD4-CD8- and CD8+ T cell subsets (Supplementary Figures 2C,D). Furthermore, analysis of the ratio of CD8+ frequency to FoxP3+ frequency within the CD4+ subset revealed no significant difference between patients and controls (*n* = 20) (Supplementary Figure 2E).

### Canine Melanoma Patients Demonstrated Increased Frequencies for Selected T Cell Subsets Based on CD25 Expression in Blood

**Figure 4:**
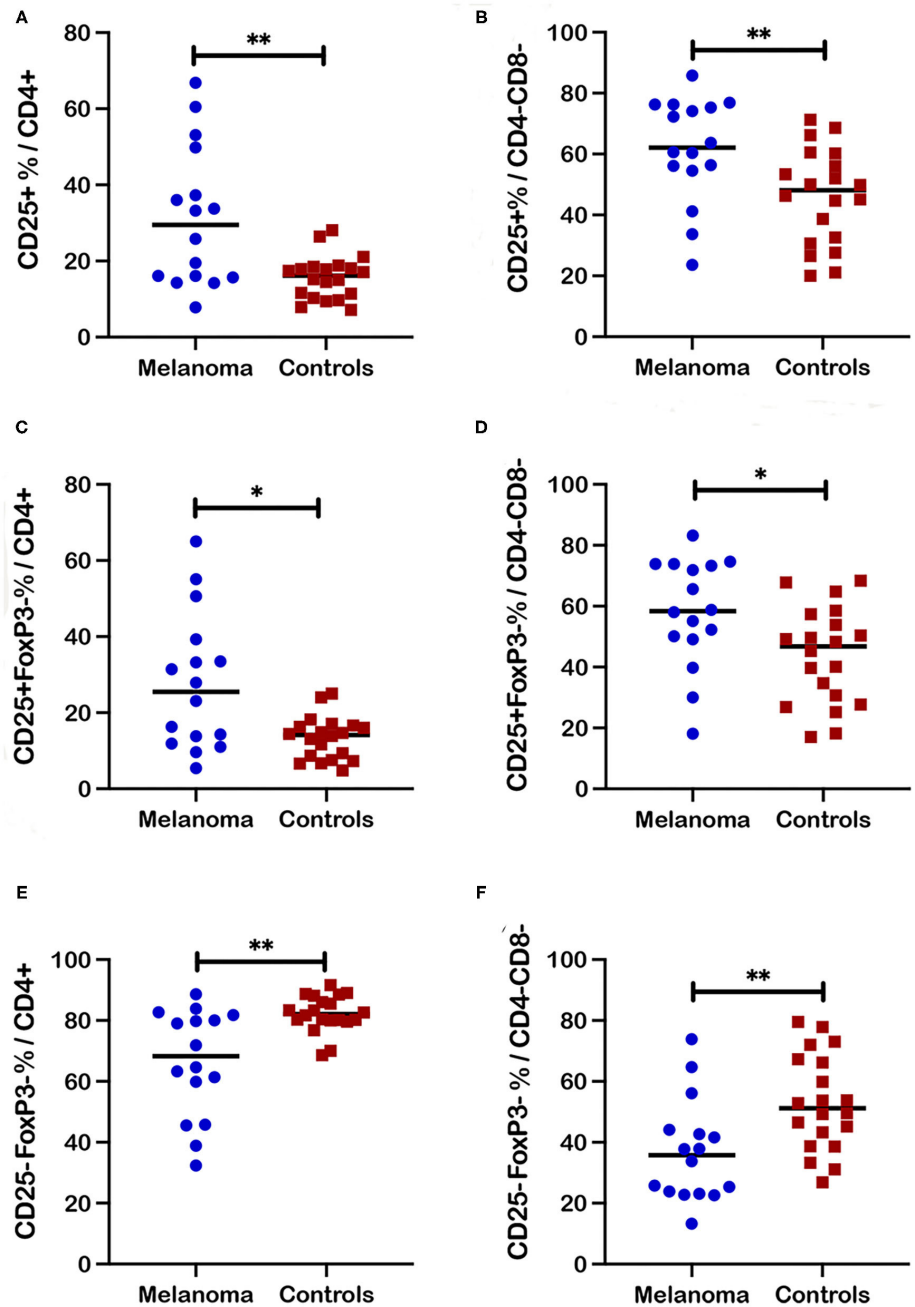
Frequencies of activated and quiescent T cell subsets in blood from canine melanoma patients and healthy controls. Median frequencies of CD25+ cells within the CD4+ **(A)** and CD4-CD8- **(B)** T cell subsets are compared between patients and healthy controls. Frequencies of CD25+FoxP3- cells within the CD4+ **(C)** and CD4-CD8- **(D)** T cell subsets are compared between patients and healthy controls. Median frequencies of CD25-FoxP3- cells within the CD4+ **(E)** and the CD4-CD8- **(F)** T cell subsets are compared between patients and healthy controls. Pair-wise analysis between patients and controls was conducted with the Mann Whitney test using GraphPad Prism software and two-tailed analysis. A single asterisk (*) denotes a *P*-value < 0.05 and two asterisks (**) denote a *P*-value < 0.01. *P*-values < 0.05 are considered significant.

CD25 as the alpha chain of the trimeric IL-2 receptor is considered a critical marker for T cell activation that is associated not only with regulatory but other activated T cell subsets. Significantly increased frequencies of CD25+ cells that include both FoxP3+ and FoxP3- populations, were observed for CD4+ (*p* = 0.01) and CD4-CD8- (*p* = 0.0038) T cell subsets in blood from canine melanoma patients (*n* = 16) compared to healthy controls (*n* = 20) (Figures 4A,B). Similarly, frequencies of CD25+FoxP3- cells were also significantly increased for the CD4+ (*p* = 0.017) and CD4-CD8- (*p* = 0.012) T cell subsets (Figures 4C,D). In contrast, frequencies of a putative quiescent phenotype CD25-FoxP3- were significantly decreased in patients compared to controls for both the CD4+ (*p* = 0.002) and CD4-CD8- (*p* = 0.0035) T cells (Figures 4E,F).

Expression of the proliferation marker Ki67 was also analyzed for multiple T cell subsets in blood from canine melanoma patients (*n* = 16) and healthy controls (*n* = 10). Ki67+ frequencies for both CD4+ and CD4-CD8- T cells within the CD3+ population were not significantly different between patients and controls (Supplementary Figures 3A,B). Frequencies of Ki67+ cells within regulatory (FoxP3+ and CD25+FoxP3+) CD4+ subsets (Supplementary Figures 3C,D) and CD4-CD8- T cell subsets (Supplementary Figures 3E,F) were also not significantly different. Absence of statistical significance between patients and controls was also observed for activated (CD25+FoxP3-) CD4+ and CD4-CD8- T cell subsets (Supplementary Figures 4A,B) and quiescent (CD25-FoxP3-) CD4+ and CD4-CD8- T cell subsets (Supplementary Figures 4C,D). Lastly, in contrast to CD4+ and CD4-CD8- T cell subsets, significant differences in frequencies for any activation/proliferation phenotypes including granzyme B+, CD25, Ki67, or CD25+FoxP3-, were not observed for the CD8+ T cell subset in blood for patients compared to controls (Supplementary Figures 5A–D). Frequencies in the CD25-FoxP3- quiescent population within the CD8+ T cell subset were also not significantly different between patients and controls (Supplementary Figure 5E).

### Canine Melanoma Patients Exhibited Two Unique Phenotypes for Interferon-γ T Cell Responses to ConA Stimulation

**Figure 5:**
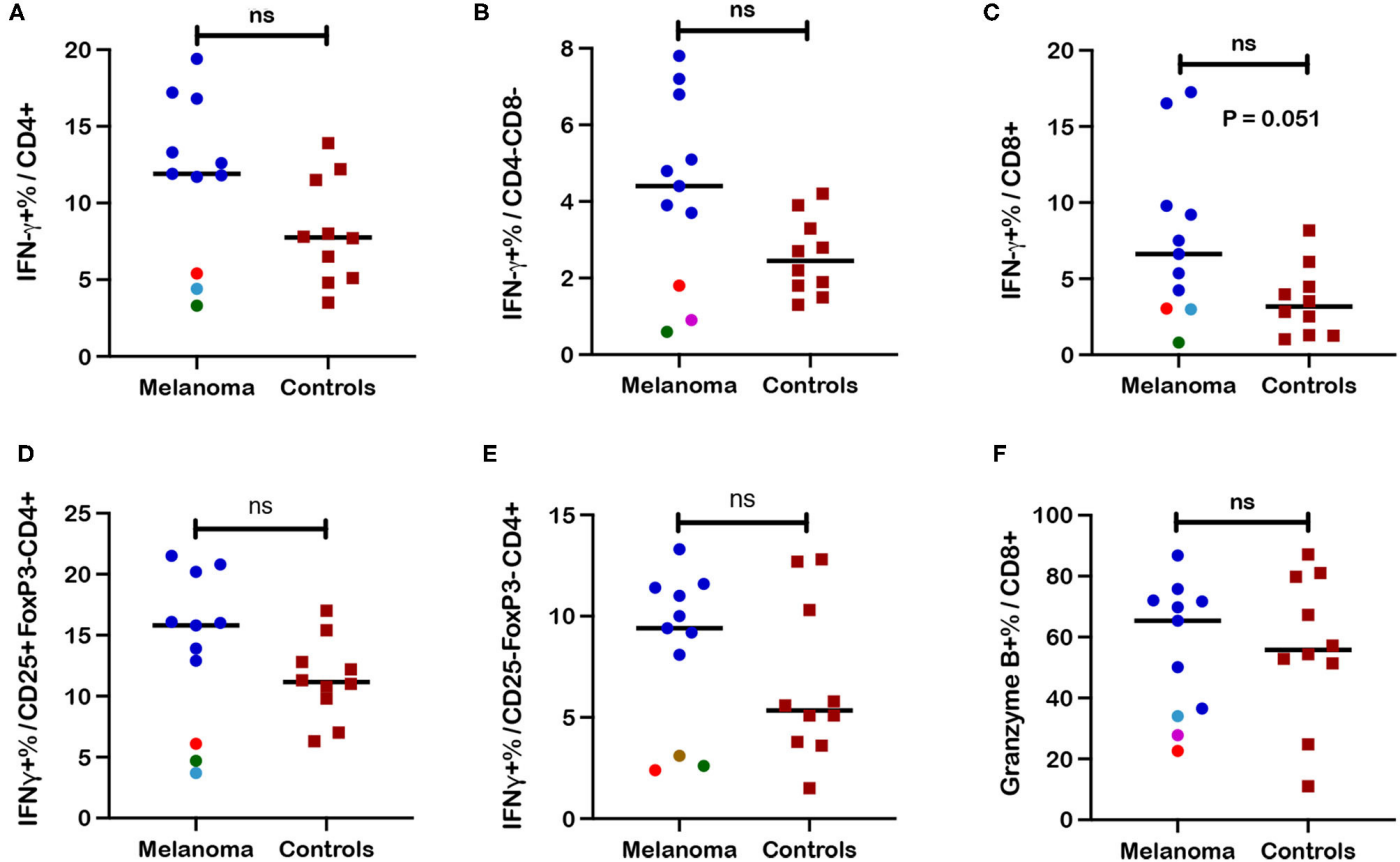
Frequencies of interferon-γ+ (IFN-γ) and granzyme-B+ T cell subsets after ConA stimulation in blood from canine melanoma patients and healthy controls. Median frequencies of IFN-γ+ cells after ConA stimulation were determined in **(A)** CD4+, **(B)** CD4-CD8-, and **(C)** CD8+ T cells by intracellular cytokine staining (ICS) and flow cytometry and compared between patients and healthy controls. Median frequencies of INF-γ+ cells after ConA stimulation are also shown for **(D)** CD25+FoxP3- (activated) and **(E)** CD25-FoxP3- (quiescent) CD4+ T cells and compared between patients and controls. **(F)** Induction of granzyme B after ConA stimulation is represented by median frequencies of granzyme B+ cells within the CD8+ T cell subset and compared between patients and controls. Colored dots represent outlier patients with green indicating subject P10, red indicating subject P11, purple indicating subject P12, turquoise indicating subject P14, and brown representing P15. Values described for IFN-γ+ frequencies reflect values for stimulated cells after subtraction of values for unstimulated cells. Values described for granzyme B+ frequencies do not reflect subtraction of values for unstimulated cells. Pair-wise analysis between patients and controls was conducted with the Mann Whitney test using GraphPad Prism software and two-tailed analysis. “ns” denotes not significant.

IFN-γ responses to ConA stimulation were measured as frequencies of IFN-γ+ cells within each T cell subset after stimulation of PBMC. IFN-γ expression after stimulation was utilized as a functional assessment of circulating T cells in canine melanoma patients (*n* = 11) and as another marker for differentiating the T cell immunophenotype of canine melanoma patients from healthy controls (*n* = 10). Although median values for IFN-γ+ frequencies were higher for CD4+, CD4-CD8-, and CD8+ T cell subsets (Figures 5A–C) in patients compared to controls, statistical differences were not observed between patients and controls, although a trend toward significance (*p* = 0.051) was observed for CD8+ T cells. Median values for IFN-γ+ cell frequencies were also higher for CD25+FoxP3- (activated phenotype) and CD25-FoxP3- (quiescent phenotype) populations within the CD4+T cell subset (Figures 5D,E) in patients compared to controls, but differences were not statistically different. Interestingly five patients (P10, P11, P12, P14, P15) demonstrated lower IFN-γ+ cell frequencies compared to other patients, for at least one T cell subset including CD4+, CD4-CD8-, CD25+FoxP3-CD4+, and CD25-FoxP3-CD4+ populations. For each subset, three of these five patients formed a cluster showing frequencies that were significantly lower (*p* = 0.012 for all four subsets) compared to other patients. Patients P10 and P11 demonstrated lower frequencies for all four subsets, with P14 showing lower frequencies for CD4+ and CD25+FoxP3-CD4+ populations, P12 for the CD4-CD8- population, and P15 for the CD25-FoxP3-CD4+ population. One patient (P10) was diagnosed with melanoma with the primary site unknown and the remaining four patients were characterized by oral tumors (Supplementary Table 1). The only other commonality for these patients was evidence of metastasis at time of diagnosis for three of the five patients (P10-12; Supplementary Table 1). These data revealed two unique phenotypes for T cell IFN-γ response to polyclonal stimulation by ConA by patient T cells that characterized patients as normal/high vs. low responders. IFN-γ+ cell frequencies for CD25+FoxP3- and CD25-FoxP3- subpopulations of other T cell subsets are not described due to extremely low events detected by flow cytometric analysis. Finally, analysis of granzyme B expression, an activation marker for CD8+ T cells after ConA stimulation, did not reveal statistical differences between frequencies of granzyme B+ CD8+ T cells after ConA stimulation between patients and healthy controls (Figure 5F). Three of the five low responder patients also demonstrated lower frequencies of either IFN-γ+ (Figure 5C) or granzyme B+ cells (Figure 5F) within the CD8+ T cell subset after ConA stimulation, although clustering of these patients from other patients was not as striking.

### Canine Melanoma Patients Demonstrated Increased Frequencies of Multiple T Cell Subsets Based on CD25 Expression in Blood After ConA Stimulation

**Figure 6:**
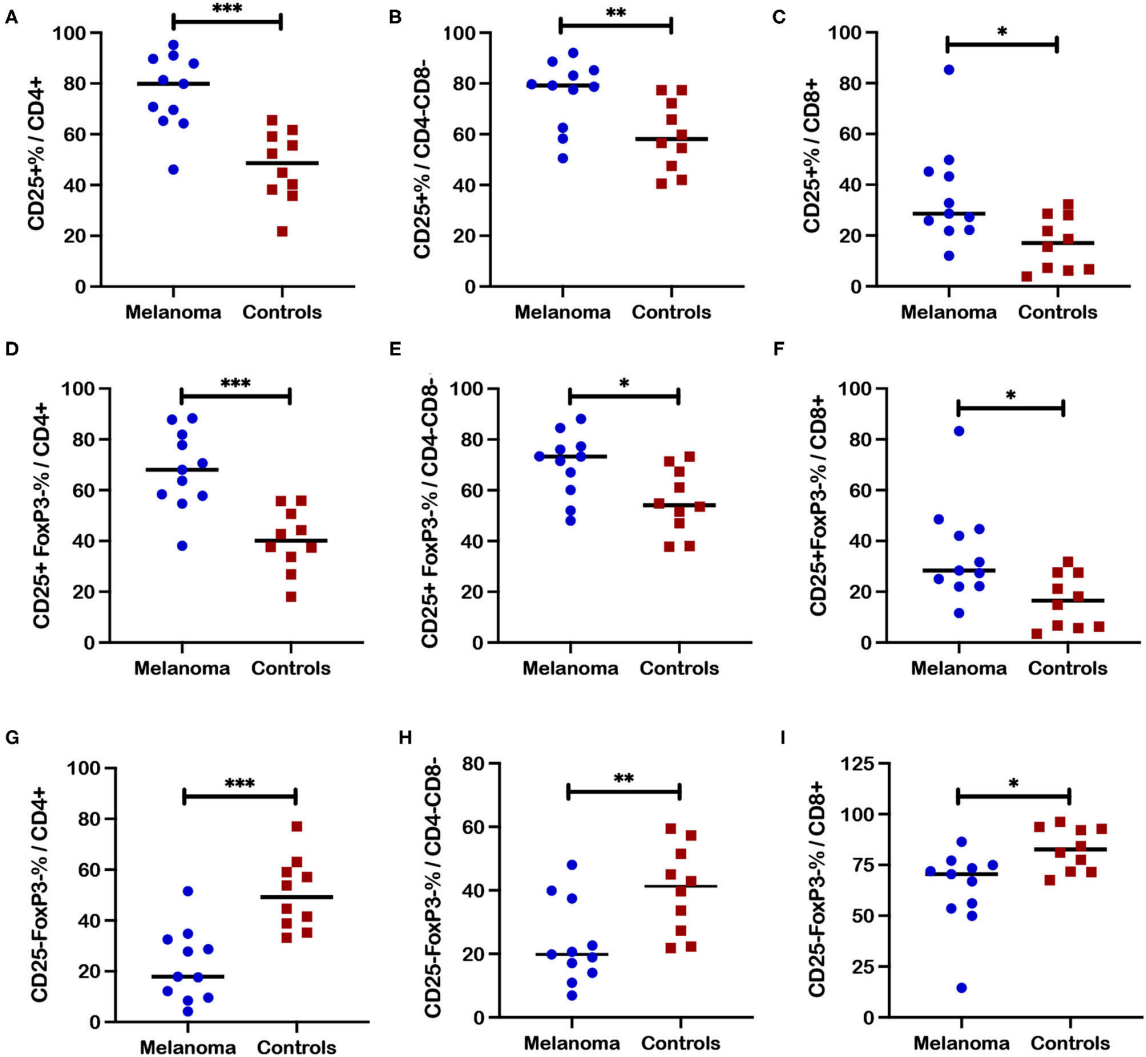
Frequencies of CD25+ T cell subsets after ConA stimulation in blood from canine melanoma patients and healthy controls. Median frequencies of CD25+ cells after ConA stimulation were determined in **(A)** CD4+, **(B)** CD4-CD8-, and **(C)** CD8+ T cells and compared between patients and healthy controls. Median cell frequencies after ConA stimulation are next shown for the activated phenotype CD25+FoxP3- within **(D)** CD4+, **(E)** CD4-CD8-, and **(F)** CD8+ T cells and compared between patients and controls. Median cell frequencies after ConA stimulation are shown for T cell phenotype CD25-FoxP3- within **(G)** CD4+, **(H)** CD4-CD8-, and **(I)** CD8+ T cell populations and compared between patients and controls. Values described for CD25+ frequencies do not reflect subtraction of values for unstimulated cells. Pair-wise analysis between patients and controls was conducted with the Mann Whitney test using GraphPad Prism software and two-tailed analysis. A single asterisk (*) denotes a *P*-value < 0.05; two asterisks (**) denote a *P*-value < 0.01; and three asterisks (***) denote a *P*-value < 0.001. *P*-values < 0.05 are considered significant.

Frequencies of CD25 expression in different T cell subsets after ConA stimulation (Figure 6) revealed patterns very similar to those observed for unstimulated T cells as shown in Figure 4 with the exception that significant differences between patients and controls were also revealed for the CD8+ T cell subset after stimulation. Significantly higher frequencies of CD25+ cells within the CD4+ (*p* = 0.0003), CD4-CD8- (*p* = 0.0045), and CD8+ (*p* = 0.011) T cell subsets were observed after ConA stimulation in patients compared to healthy controls (Figures 6A–C). Significantly higher frequencies were also observed for the activated CD25+FoxP3- population within the CD4+ (*p* = 0.0003), CD4-CD8- (*p* = 0.017), and CD8+ (*p* = 0.013) T cell subsets after ConA stimulation for patients compared to controls (Figures 6D–F). Also similar to the results for unstimulated cells, significantly lower frequencies for the quiescent CD25-FoxP3- population within the CD4+ (*p* = 0.0002), CD4-CD8- (*p* = 0.008), and CD8+ (*p* = 0.01) T cell subsets after ConA stimulation were observed for patients compared to controls (Figures 6G–I). In contrast, no significant differences between patients and controls were observed for frequencies of regulatory FoxP3+ and CD25+FoxP3+ cells within the CD4+ (Supplementary Figures 6A,B) or the CD4-CD8- (Supplementary Figures 6C,D) T cell subsets post ConA stimulation. Event numbers were too low for analysis of regulatory cell populations with CD8+ T cell subset.

Proliferation responses based on Ki67+ cell frequencies are not reported for T cell subsets as no induction of Ki67 expression was observed for either patients or controls after ConA stimulation. The absence of such responses was likely due to the need for an extended time frame in culture for restoration of proliferative function for cryopreserved lymphocytes (55, 56), as well as an extended time frame of ConA stimulation for optimal induction of Ki67: both conditions were not accommodated by our experimental protocol.

### Significantly Higher Frequencies of FoxP3+ T Cells Were Detected in Tumor Associated Cells Compared to Blood

**Figure 7:**
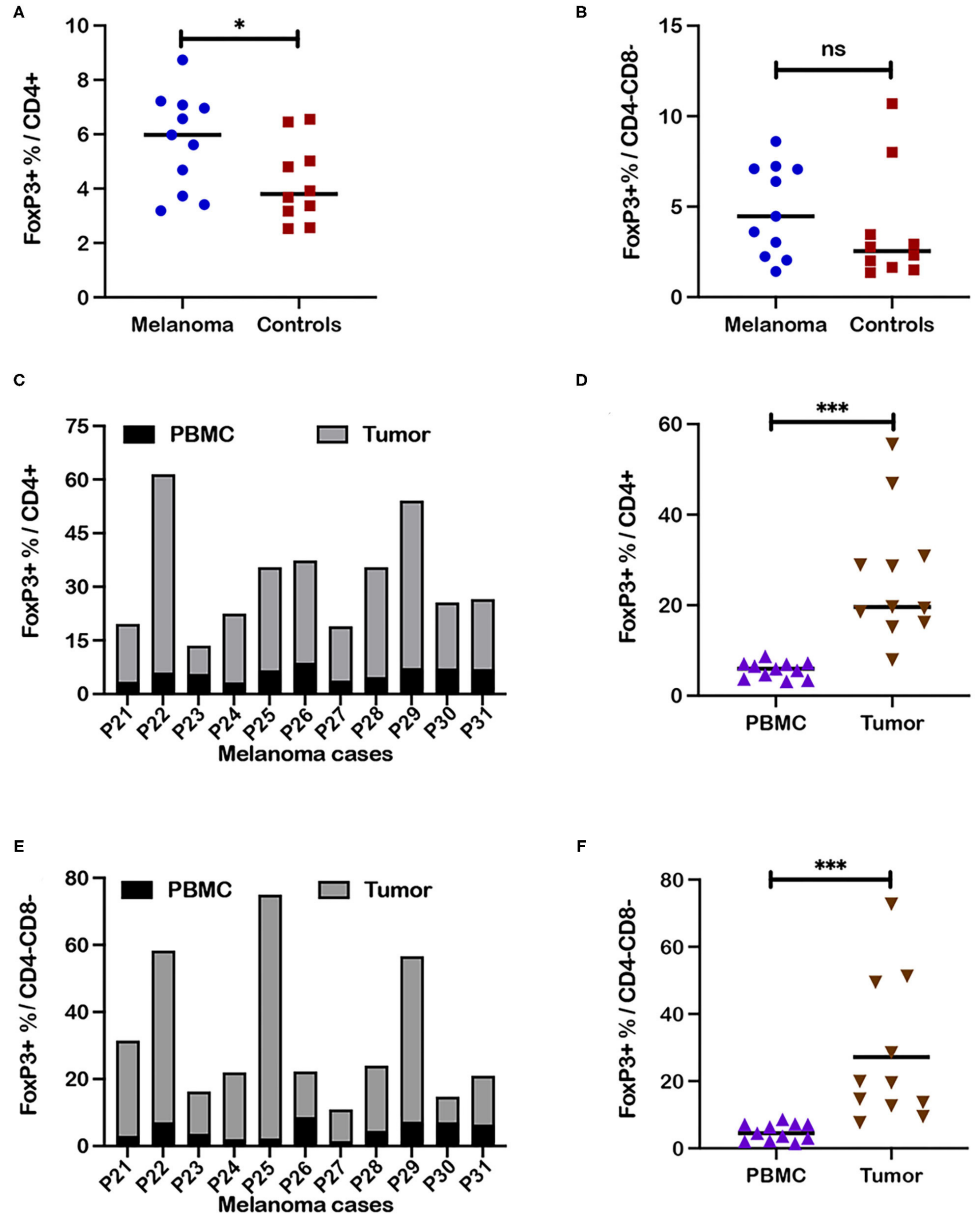
Comparison of frequencies of regulatory T cell subsets in tumor associated cells and blood in canine melanoma patients. PBMC isolated from blood and tumor associated cells from a separate cohort of 11 canine melanoma patients were assessed for T cell phenotypes by flow cytometry. **(A)** Median frequencies of FoxP3+ within CD4+ and **(B)** CD4-CD8- T cell populations in blood were compared between patients of this patient cohort and healthy controls. **(C)** Each stacked bar reflects frequencies of FoxP3+ cells within the CD4+ T cell subset in blood vs. tumor associated cells for a single patient. A comparison of frequencies assessed for blood and tumor associated cells is shown for all patients. **(D)** Median frequencies of FoxP3+ cells within the CD4+ T cell subset are compared between PBMC and tumor as a grouped analysis for the same patients. **(E)** A stacked bar analysis of frequencies of FoxP3+ cells within the CD4-CD8- T cell subset in blood vs. tumor associated cells from each patient is shown for all patients. **(F)** Median frequencies of FoxP3+ cells within the CD4-CD8- T cell subset are compared between PBMC and tumor as a grouped analysis for the same patients. Pair-wise analysis between frequencies for patients and controls was conducted with the Mann Whitney test and between PBMC and tumor associated cells using the Wilcoxon matched-pairs signed rank test, using GraphPad Prism software and a two-tailed analysis. “ns” denotes not significant. A single asterisk (*) denotes a *P*-value < 0.05; three asterisks (***) denote a *P*-value < 0.001. *P*-values < 0.05 are considered significant.

Fresh PBMC and tumor associated cells were isolated from a separate cohort of canine melanoma patients (P21-31; Supplementary Tables 1, 2) and tested by an abbreviated flow cytometry panel that included interrogation for CD3+ T cell subsets (CD4+, CD4-CD8-, and CD8+), FoxP3 and granzyme B (Supplementary Figure 7). FoxP3+ frequencies within the CD4+ T cell subset in fresh PBMC for this patient cohort (*n* = 11) were higher compared to fresh PBMC isolated from healthy controls (*n* = 10; C14-23) (*p* = 0.043) (Figure 7A; Supplementary Figure 7B), although the difference was modest as shown for other melanoma patients (Figure 3B). Although median frequencies for FoxP3+ cells within the CD4-CD8- T cell subset were higher for patients compared to controls, a significant difference was not detected (Figure 7B). A comparison of frequencies of FoxP3+ cells within the CD4+ T cell subset for tumor associated cells and blood (PBMC) for each patient revealed a significantly higher frequency of FoxP3+ cells in tumor vs. blood for each patient (Figures 7C,D) (*p* = 0.001). A similar result was noted for comparison of FoxP3+ cell frequencies within the CD4-CD8- T cell subset with higher frequencies detected in tumor associated cells compared to blood for each patient (Figures 7E,F) (*p* = 0.001). Comparison of frequencies of CD4+, CD4-CD8-, and CD8+ T cell subsets between PBMC and tumor associated cells did not reveal significant differences (Supplementary Figures 8A–F). Significant differences in frequencies of granzyme B+ cells within the CD8+ T cell subset were also not detected between tumor associated cells and PBMC for each patient (Supplementary Figures 8G,H). Importantly intratumoral frequencies of FoxP3+ cells within the CD4+ and CD4-CD8- subsets were strikingly higher compared to frequencies in PBMC.

### Frequencies of Regulatory, Activation, and Proliferation Markers Differed Between Specific T Cell Subsets in Blood for Healthy Controls

**Figure 8:**
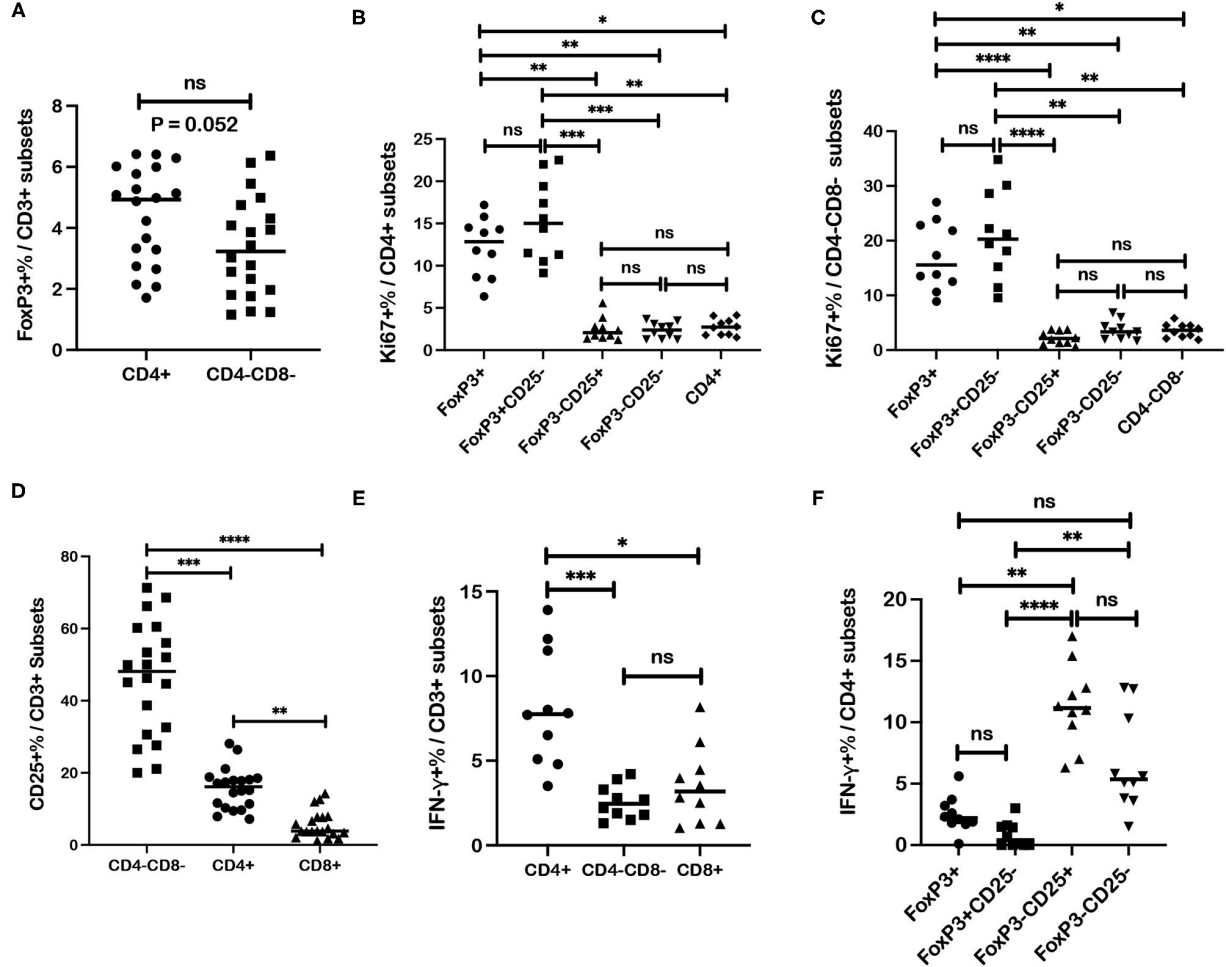
Associations between regulatory and activation markers for specific T cell subsets in healthy dog PBMC. **(A)** Median frequencies of FoxP3+ cells are compared between CD4+ and CD4-CD8- T cell subsets. **(B)** Median frequencies of Ki67+ cells within different subsets of CD4+ T cells including regulatory (FoxP3+; CD25-FoxP3+), activated (CD25+FoxP3-) and quiescent (CD25-FoxP3-) populations, are compared. **(C)** Median frequencies of Ki67+ cells within different subsets of CD4-CD8- T cells including regulatory (FoxP3+), activated (CD25+FoxP3-) and quiescent (CD25-FoxP3-) populations, are compared. **(D)** CD4+, CD4-CD8-, and CD8+ T cells subsets are compared for median frequencies of CD25+ cells. **(E)** Median frequencies of interferon-γ (IFN-γ)+ cells after ConA stimulation were compared between CD4+, CD4-CD8-, and CD8+ T cells subsets. **(F)** Median frequencies of IFN-γ+ cells after ConA stimulation were compared between different CD4+ T cell subsets including regulatory (FoxP3+; CD25-FoxP3+), activated (CD25+FoxP3-) and quiescent (CD25-FoxP3-) populations. Values described for IFN-γ+ frequencies reflect values for stimulated cells after subtraction of values for unstimulated cells. Comparisons of frequencies between three more subsets were performed with a Kruskal-Wallis test with Dunn's *post-hoc* test for multiple comparisons. Pair-wise analysis between frequencies of two subsets **(A)** was conducted with the Mann Whitney test and two-tailed analysis. All analyses used GraphPad Prism software. “ns” denotes not significant. A single asterisk (*) denotes a *P*-value < 0.05; two asterisks (**) denote a *P*-value < 0.01; three asterisks (***) denote a *P*-value < 0.001 and four asterisks (****) denote a *P*-value < 0.0001. *P*-values < 0.05 are considered significant.

Data generated from these studies of canine melanoma patients afforded an opportunity to analyze different canine T cell subsets and subpopulations for specific T cell markers based on healthy control dogs. An interesting CD4-CD8- CD3+ T cell subset was reported and characterized previously to express regulatory markers including FoxP3 and CD25 (44). Our results for 20 healthy control dogs also show FoxP3 expression by CD4-CD8- T cells although frequencies of FoxP3+ cells for this subset are lower compared to CD4+ cells with a difference trending for significance (*p* = 0.052) (Figure 8A). Regardless frequencies of FoxP3+ cells for CD4-CD8- T cells (median = 3.23%) were still comparable to CD4+ cells (median = 4.93%) and significantly higher than those assessed for CD8+ T cells (median = 0.54%) (*p* < 0.0001).

Analysis of healthy controls (*n* = 10) revealed higher Ki67+ cell frequencies within the FoxP3+CD4+ T cells compared to frequencies within CD25+FoxP3- (*p* = 0.001) and CD25-FoxP3- (*p* = 0.002) subpopulations of CD4+ T cells and also compared to Ki67+ cell frequency with the total CD4+ T cell subset (*p* = 0.018). Ki67+ cell frequencies were also higher in the CD25-FoxP3+CD4+ population compared to CD25+FoxP3-CD4+ (*p* = 0.0001) and CD25-FoxP3-CD4+ (*p* = 0.002) populations and also compared to Ki67+ cell frequency within the total CD4+ T cell subset (*p* = 0.024). Ki67+ cell frequencies were not significantly different between regulatory populations FoxP3+CD4+ and CD25-FoxP3+ CD4+ T cells, or between different FoxP3-CD4+ T cell populations (CD25+FoxP3- and CD25-FoxP3-) or compared to Ki67+ cell frequency within the total CD4+ T cell subset (Figure 8B). Similar results were shown for the CD4-CD8- subset where Ki67+ cell frequencies detected within the FoxP3+CD4-CD8- T cell subset were higher compared to Ki67+ cell frequencies for CD25+FoxP3- (*p* < 0.0001) and CD25-FoxP3- (*p* = 0.0097) populations of the CD4-CD8- T subset, and also compared to Ki67+ cell frequency with the total CD4-CD8- T cell subset (*p* = 0.015). Ki67+ cell frequencies were also higher in CD25-FoxP3+CD4-CD8- population compared to CD25+FoxP3- (*p* < 0.0001) and CD25-FoxP3- (*p* = 0.0022) populations of CD4-CD8- T cells, and also compared to Ki67+ cell frequency with the total CD4-CD8- T cell subset (*p* = 0.0035). Ki67+ cell frequencies were not significantly different between FoxP3+ and CD25-FoxP3+ populations within CD4-CD8- subset, or between FoxP3-CD4-CD8- T cell populations (CD25+FoxP3- and CD25-FoxP3-) or between FoxP3-CD4-CD8- T cell subsets and the total CD4-CD8- T cell subset (Figure 8C). These results overall show higher Ki67+ cell frequencies within FoxP3+ populations of both CD4+ and CD4-CD8- T cell subsets. Assessment of similar populations within the CD8+ T cell subset was not performed due to extremely low numbers of events detected by flow cytometric analysis for this T cell subset.

Frequencies of CD25+ cell populations within each T cell subset were analyzed and revealed a significantly higher frequency in the CD4-CD8- T cell subset compared to CD4+ (*p* = 0.0006) and CD8+ (*p* < 0.0001) T cells (Figure 8D). As expected CD25+ cell frequencies for CD4+ cells were also higher compared to the CD8+ subset (*p* = 0.004). Frequencies of activated CD25+FoxP3- cells were also higher for the CD4-CD8- subset compared to CD4+ cells (*p* = 0.0004) and CD8+ cells (*p* < 0.0001) (Supplementary Figure 9). Also as expected, frequencies of CD25+FoxP3- cells were higher in CD4+ cells compared to CD8+ cells (*p* = 0.009). These results reveal the highest frequency of activated CD25+ cells within the CD4-CD8- T cell subset followed by the CD4+ subset.

IFN-γ+ cell frequencies after ConA stimulation were higher for CD4+ T cells compared to CD4-CD8- T cells (*p* = 0.0009) and CD8+ T cells (*p* = 0.012) (Figure 8E). Differences in IFN-γ+ cell frequencies were not significant between CD4-CD8- and CD8+ T cells. Analysis of IFN-γ+ cell frequencies within FoxP3+ and FoxP3- CD4+ subsets after ConA stimulation was also performed. Significantly higher IFN-γ+ cell frequencies were detected in CD25+FoxP3-CD4+ T cells compared to regulatory FoxP3+CD4+ cells (*p* = 0.004) and CD25-FoxP3+CD4+ cells (*p* < 0.0001) (Figure 8F). IFN-γ+ cell frequencies were also higher in the CD25-FoxP3-CD4+ T cells compared to CD25-FoxP3+CD4+ cells (*p* = 0.002). Comparisons of IFN-γ+ cell frequencies between FoxP3+CD4+ and CD25-FoxP3+CD4+ subsets, FoxP3+CD4+ and CD25-FoxP3-CD4+ subsets, and CD25+FoxP3-CD4+ and CD25-FoxP3-CD4+ subsets revealed no significant differences (Figure 8F). Overall, these data show higher IFN-γ+ cell frequencies for the CD4+ T cell subset and particularly for the CD25+FoxP3-CD4+ subset with low IFN-γ+ cell frequencies found in FoxP3+ CD4 subsets. Also of note, IFN-γ+ cell frequencies detected with the CD4-CD8- subset were comparable to those detected in the CD8+ subset and distinguishes one property shared by the CD4-CD8- and CD8+ subsets.

## Discussion

T cell immunophenotyping studies described herein were predicated on the hypothesis that canine melanoma patients exhibit a T cell immune profile that is different from healthy control pet dogs. A secondary hypothesis states that frequencies of circulating T cell subsets may not match intratumoral frequencies and that blood vs. tumor T cell immunophenotypes will differ. Findings by PCA indeed determined that canine melanoma patients display a T cell immunophenotype that is unique from healthy pet dogs. These findings were further supported by analysis of frequencies of individual T cell immunotypes in patients and healthy controls. Key findings revealed increased frequencies of circulating regulatory FoxP3+ T cell subsets and activated CD25+FoxP3- T cell subsets in canine patients compared to healthy controls. Furthermore, T cell function measured by IFN-γ responses to ConA stimulation in PBMC distinguished canine melanoma patients into two groups with a larger group demonstrating a higher frequency of IFN-γ+ cells indicating a competent and possibly inflated functional response. A second smaller group of patients showed a considerably weaker response suggestive of a dysregulated response. Another key finding was the dichotomy determined between frequencies of FoxP3+ CD4+ and FoxP3+ CD4-CD8- cells in blood compared to tumor where frequencies of FoxP3+ cells were significantly higher in tissue than detected in circulation. Lastly, the CD4-CD8- T cell population frequently displayed an immunophenotype similar to CD4+ T cells in blood and tumor with the exceptions of amplified CD25 expression and an IFN-γ response that was more similar to CD8+ T cells. These findings although not surprising, collectively characterized a T cell immunophenotype that clearly distinguished canine melanoma patients from healthy controls and suggested immune pathways for further investigation.

Previous reports have described increased frequencies of circulating and intratumoral Tregs defined either as FoxP3+CD4+ or CD25+FoxP3+CD4+ T cell subsets for canine melanomas by either flow cytometry analysis (57–61) or immunohistochemistry (IHC) (60–63). Our findings also revealed significantly increased frequencies for either FoxP3+ or CD25+FoxP3+ populations in either CD4+ or CD4-CD8- T cell subsets in PBMC from patients compared to controls, although differences were fairly small and CD4+ T cell frequencies were actually lower in patients. A finding of greater interest was the significantly higher frequency of FoxP3+ cells within both the CD4+ and CD4-CD8- T cell subsets in tumor associated cells compared to corresponding patient blood samples from a small case cohort, confirming the importance of examining the local tumor environment. Although few in number, other reports have described similar observations of increased frequencies of FoxP3+ cells detected within either tumor or draining lymph node for canine melanoma patients assessed by flow cytometry (57, 60) or by IHC Treg (61). Although intratumoral Tregs have been reported to confer immunosuppression by multiple mechanisms including inhibition of anti-tumor effector responses and to promote disease progression for a wide range of human cancers (64, 65) there are conflicting reports as to whether FoxP3+ tumor-infiltrating lymphocytes (TILs) confer a negative prognosis for human cutaneous melanoma (64). Two reports describing Tregs within TILs for canine melanoma concluded that higher frequencies of intratumoral Treg were a negative prognostic factor (60, 63) whereas a more recent report (61) did not find intratumoral Treg frequency to associate with more aggressive disease. Our findings on a small canine melanoma cohort did not find significant differences between frequencies of other T cell subsets (CD4+, CD8+, and CD4-CD8-) or frequencies of granzyme B+ CD8+ T cells in blood vs. tumor. Furthermore, the canine cohort was insufficient in case number to determine an association of intratumoral Treg frequency and disease outcome. Future studies with larger patient cohorts and flow cytometry panels with additional markers along with corresponding IHC analysis will be necessary to determine the relevance of the large Treg frequencies in tumor associated cells as observed in this study and to identify intratumoral T cell phenotypes as correlates for prognosis and potential immunotherapeutics.

CD25 proved to be a major marker of interest in characterizing T cell immunophenotypes in canine melanoma patients. CD25 expression has classically been defined as a Treg marker when co-expressed with FoxP3+ in CD4+ T cells (66, 67). In contrast to reports for frequencies of healthy human CD25+ CD4+ T cell (68, 69), our studies revealed a higher frequency of this subset within both healthy controls and patients which proved to include both FoxP3- as well as FoxP3+ cell populations. Other reports describing CD25+ cell frequencies within the CD4+ T cell subset in healthy dogs also revealed similar or higher frequencies compared to our current studies (44, 70, 71). Our results also revealed increased frequencies of the CD25+ cells for both CD4+ and CD4-CD8- T cell subsets in canine melanoma patients compared to healthy dog controls. A more interesting finding related to the significant frequencies of CD25+FoxP3- cell population within both CD4+ and CD4-CD8- T cell subsets for healthy controls and patients. Furthermore, frequencies of the CD25+FoxP3- cell population in both T cell subsets were significantly higher in patients. Reports specifically describing CD25+FoxP3- cells within either CD4+ and CD4-CD8- T cell subsets for different species are few. However, CD25 as the alpha chain of the trimeric IL-2 receptor is considered a critical marker for T cell activation for not only regulatory, but also recently activated effector and antigen-experienced or resting memory T cells in mice and humans (69, 72–76). Moreover, frequencies of both CD25+ and CD25+FoxP3- populations were increased for all T cell subsets including CD8+ T cells after stimulation by ConA, again with patients again showing significantly higher frequencies compared to healthy controls. Conversely no differences in frequencies of FoxP3+ or CD25+FoxP3+ populations for either CD4+ or CD4-CD8- T cell subsets after stimulation by ConA were observed between patients and controls. These findings would suggest that the CD25+FoxP3- population within each T cell subset, particularly the CD4+ and CD4-CD8- subsets, represented an activated population primed for expansion upon polyclonal stimulation with a heightened response shown by patients.

Given that higher frequencies of the activated CD25+FoxP3- population were higher in patients, lower frequencies of the putative quiescent CD25-FoxP3- population were detected within both CD4+ and CD4-CD8- subsets for patients compared to healthy controls in unstimulated and ConA-stimulated cells. Stimulated CD8+ T cells also revealed lower frequencies for the CD25-FoxP3- population in patients compared to controls. Based on the absence of expression by FoxP3 (considered to be a regulatory and activation marker) and absence of activation marker CD25, this population was tentatively considered a “quiescent” T cell population. However, both CD25+FoxP3- and CD25-FoxP3- populations are most likely heterogenous populations and the CD25-FoxP3- population will include both naive and memory populations. Therefore, these populations will require further interrogation by memory and other activation markers for an accurate identification and determination of specific sub-populations that may be accountable for differences between canine melanoma patients and healthy controls. It is important to note that proliferation marker Ki67 did not clearly distinguish any T cell subsets or sub-populations for differences in frequencies between patients and controls. Similarly, granzyme B as an activation and exhaustion marker for CD8+ T cells, did not detect differences in frequencies of granzyme B+ CD8+ cells between patients and healthy controls for either unstimulated or stimulated cells. However, patients demonstrated a large variation in values for frequencies of granzyme B+ cells compared to controls and revealed selected outlier patients showing very high frequencies. Further examination of granzyme B as a CD8+ T cell phenotype will be warranted in larger patient cohorts.

T cell dysfunction or T cell exhaustion is a well-recognized outcome of chronic antigenic stimulation and cancer and may result in a progressive loss of T cell functions including proliferation, cytokine release and cytolytic activity. This is a consequence of the actions of multiple inhibitory receptors including PD1 and CTLA-4 that are induced by chronic antigenic stimulation or inhibitory receptor ligands expressed by tumor cells (39). IFN-γ is a type 2 pleiotropic interferon that displays antiproliferative, anti-angiogenic and pro-apoptotic activity against tumor cells through multiple complex mechanisms (39, 77). IFN-γ is produced predominantly by activated CD4+ T cells (Th1), CD8+ T cells, γδ T cells, and natural killer (NK) cells and may also demonstrate suppressive effects on anti-tumor immune responses by induction of multiple immunoregulatory factors on tumor cells including indoleamine-2,3-dioxygenase (IDO) and PDL1. To assay for T cell dysfunction in a small cohort of canine melanoma patients, induction of IFN-γ expression in response to mitogen (ConA) stimulation was measured in PBMC by ICS in both patients and healthy controls. Increased IFN-γ+ cell frequencies within T cell subsets after ConA stimulation were observed for patients compared to healthy controls for all subsets. However, differences were not statistically significant although a trend for a significant difference between patients and controls was noted for CD8+ T cells. Of note, patients revealed two different phenotypic responses with five out of 11 patients showing IFN-γ+ cell frequencies much lower than those of other patients for at least one or more CD4+ or CD4-CD8- T cell subsets. These findings suggested that IFN-γ release as a T cell function was spared in a moderate proportion of patients (6/11) whereas this specific function was affected in a subset of patients. Our findings therefore slightly differed from results of the one other report describing IFN-γ release after mitogen stimulation in canine melanoma patients which revealed significantly lower frequencies of IFN-γ+ cell frequencies for patients compared to controls (58). Conditions in this previous report utilized PMA and ionomycin for stimulation and therefore direct comparisons of the two studies are not possible. The patient cohort in our study is too small to determine the relevance of this dysfunctional response to disease progression or as a prognostic marker. In summary, assay of additional T cell functions including induction of release of other cytokines and perhaps using different stimulation protocols, should be examined in larger patient cohorts in future studies to further characterize T cell functions as biomarkers in canine melanoma patients.

Characterization of regulatory (FoxP3+), activation (CD25+FoxP3-), proliferation (Ki67+), and functional (IFN-γ+) markers for different canine T cell subsets was not a goal of these studies. However, analysis of healthy dog controls within these studies allowed this type of analysis for canine T cells. The CD4-CD8- T cell subset in dogs was described previously as an activated subset that demonstrates significantly higher frequencies of CD25+ cells (44) compared to CD4+ and CD8+ T cells. This finding was also observed in our studies with a large range of values for frequencies of CD25+ cells within the CD4-CD8- T cell subset, but with a median frequency well above that of CD4+ T cells. Likewise, higher frequencies of CD25+FoxP3- cells were detected for the CD4-CD8- T cell subset when compared to CD4+ and CD8+ subsets. No significant difference between frequencies of FoxP3+ cells within the CD4-CD8- and CD4+ subsets was detected by our results or by Rabiger et al. Moreover, CD4+ and CD4-CD8- T cell subsets demonstrated similar patterns for increased Ki67+ cell frequencies within FoxP3+ populations compared to FoxP3- populations including the CD25+FoxP3- population. Higher frequencies of Ki67+ cells within circulating Tregs compared to FoxP3-CD4+ T cells have also been reported in humans along with the observation that Treg populations are highly proliferative (78, 79). Furthermore, another report identified Ki67+ cell frequencies within the Treg as a negative prognostic factor (80) for human ovarian cancer. However, a comparison of IFN-γ+ cell frequencies between T cell subsets after ConA stimulation revealed frequencies for the CD4-CD8- subset were comparable to CD8+ T cells and significantly lower than those for CD4+ T cells. This finding revealed a property that distinguished the CD4-CD8- T cells from CD4+ T cells. With the exception of IFN-γ expression, our studies suggest that canine CD4-CD8- T cells share similar phenotypes with CD4+ T cells although the expression of CD25, typically a property of CD4+ T cells, was significantly higher for this subset. It is important to note that the CD3 monoclonal antibody used in these studies detects the epsilon domain of CD3 (CD3ε) and therefore detects both TCR-αβ+ and TCR-γδ+ T cell subsets. Furthermore, the CD3ε domain has been reported to be expressed on a small subset of human natural killer (NK) cells (81–83), whereas canine NK cells have been characterized as TCR-αβ+ CD3+CD5-low cells (84–87). Accordingly, the CD4-CD8- CD3+ population detected in our studies may include multiple populations including both TCR-αβ+ and TCR-γδ+ T cells, NK cells and possibly NKT cells. Gene expression analysis of the canine T cells subsets will be necessary to further distinguish CD4-CD8- T cell from the conventional CD4+ and CD8+ subsets. Lastly, analysis of IFN-γ+ frequencies after stimulation within CD4+ T cell subsets revealed frequencies within the CD25+FoxP3- population, that were significantly higher compared to FoxP3+ populations and also higher than CD25-FoxP3- CD4+ cells. These findings were not unexpected based on other reports describing restricted IFN-γ expression by Tregs in other species and instead Tregs typically secrete regulatory immunomodulators including IL-10 and Transforming growth factor b (TGF-b) (88–90). However, these results further defined CD25+FoxP3- T cells as an activated population unique from canine Treg, and also as a T cell population of interest for further investigation in canine cancer by both gene expression analysis and an expanded examination for additional T cell markers.

Selection of T cell markers utilized in panels for these studies was based on availability of canine cross-reactive antibodies available and also reported in the literature at the time the project was initiated. Based on reports in humans and dogs, markers for regulatory T cells (FoxP3 and CD25), proliferation/activation, (Ki67), (granzyme B), and functionality and T cell programming (IFN-γ) were feasible for a single panel and provided a diverse set of parameters for a preliminary assessment of T cell phenotyping of canine melanoma patients and controls. Limitations of the selected markers are acknowledged and additional T cell markers currently available would include eomes (34), PD-1 (25, 91, 92), T-bet and GATA-3 (44), memory markers CD62L and CD45RA (33) and additional other markers including cytokines including such as TNF-α.

The tumor and patient information compared to T cell phenotypes was examined by PCA which provided an additional strategy for addressing the basic question of whether canine melanoma patients display a T cell phenotye that is different from healthy dogs. However, PCA precludes cases where a single component of data is absent (example: mitotic index) and related to immunophenotype, exclusion of a marker for which only a limited number of samples were assayed (Ki67 missing for 50% of controls). Additionally, the canine melanoma case cohort overall was small and low in numbers for less progressive melanoma phenotypes which hindered analysis for correlations of immunophenotypes with survival or disease progression. An analysis of progression or survival was also not possible in this survey study as some of the subjects had no treatment and those that did undergo treatment had a variety of therapies. Accordingly, analysis of individual T cell phenotypes also did not reveal significant differences for different clinical parameters. Despite these limitations PCA determined an association between immunophenotype and melanoma patients with metastasis, which should be explored in further studies.

Importantly PCA determined that melanoma patients displayed a unique T cell phenotype by analysis of both unstimulated and stimulated T cell populations when compared to healthy control and provided further support of our overall hypothesis. Furthermore, findings from analysis of individual T cell immunophenotypes determined by flow cytometry revealed specific phenotypes defined by both regulatory and activation markers that distinguished canine melanoma patients and healthy controls. These results represent as survey investigation of canine melanoma patients with an array of T cell markers that has not previously been reported when tested in combination. As such, these findings will direct future assessment of a larger canine melanoma patient cohort with flow cytometry panels that accommodate additional T cell markers to further define specific T cell subsets as biomarkers for tumor stage, disease progression and response to specific immunotherapeutics.

## Data Availability Statement

The raw data supporting the conclusions of this article will be made available by the authors, without undue reservation.

## Ethics Statement

The animal study was reviewed and approved by UC Davis IACUC and Clinical Trials Review Board. Written informed consent was obtained from the owners for the participation of their animals in this study.

## Author Contributions

MK, ES, RR, and AM contributed to conception and design of the study. ES, MK, HC, HK, SW, and RC contributed to flow cytometry panel development. ES, NC, and MK performed the statistical analysis. ES and MK wrote the first draft of the manuscript. HC and NC wrote sections of the manuscript. All authors contributed to manuscript revision, read, and approved the submitted version.

## Funding

Supported in part by the Center for Companion Animal Health, School of Veterinary Medicine, University of California, Davis and by National Cancer Institute P30CA093373-14S4, U01 CA224166-0 and through the Flow Cytometry Shared Resource, National Cancer Institute P30CA093373.

## Conflict of Interest

The authors declare that the research was conducted in the absence of any commercial or financial relationships that could be construed as a potential conflict of interest.

## Publisher's Note

All claims expressed in this article are solely those of the authors and do not necessarily represent those of their affiliated organizations, or those of the publisher, the editors and the reviewers. Any product that may be evaluated in this article, or claim that may be made by its manufacturer, is not guaranteed or endorsed by the publisher.

## References

[1] Hodi FS O'Day SJ McDermott DF Weber RW Sosman JA Haanen JB. Improved survival with ipilimumab in patients with metastatic melanoma. N Engl J Med. (2010) 363:711–23. 10.1056/NEJMoa1003466 20525992 PMC3549297

[2] Keilholz U Ascierto PA Dummer R Robert C Lorigan P van Akkooi A. ESMO consensus conference recommendations on the management of metastatic melanoma: under the auspices of the ESMO Guidelines Committee. Ann Oncol. (2020) 31:1435–48. 10.1016/j.annonc.2020.07.004 32763453

[3] Dimitriou F Staeger R Ak M Maissen M Kudura K Barysch MJ. Frequency, treatment and outcome of immune-related toxicities in patients with immune-checkpoint inhibitors for advanced melanoma: results from an institutional database analysis. Cancers. (2021) 13:122931. 10.3390/cancers13122931 34208218 PMC8230729

[4] Steininger J Gellrich FF Schulz A Westphal D Beissert S Meier F. Systemic therapy of metastatic melanoma: on the road to cure. Cancers. (2021) 13:61430. 10.3390/cancers13061430 33804800 PMC8003858

[5] Camisaschi C Vallacchi V Castelli C Rivoltini L Rodolfo M. Immune cells in the melanoma microenvironment hold information for prediction of the risk of recurrence and response to treatment. Expert Rev Mol Diagn. (2014) 14:643–6. 10.1586/14737159.2014.928206 24914691

[6] Simpson RM Bastian BC Michael HT Webster JD Prasad ML Conway CM. Sporadic naturally occurring melanoma in dogs as a preclinical model for human melanoma. Pigment Cell Melanoma Res. (2014) 27:37–47. 10.1111/pcmr.12185 24128326 PMC4066658

[7] Prouteau A Andre C. Canine melanomas as models for human melanomas: clinical, histological, and genetic comparison. Genes. (2019) 10:70501. 10.3390/genes10070501 31262050 PMC6678806

[8] Rahman MM Lai YC Husna AA Chen HW Tanaka Y Kawaguchi H. Transcriptome analysis of dog oral melanoma and its oncogenic analogy with human melanoma. Oncol Rep. (2020) 43:16–30. 10.3892/or.2019.7391 31661138 PMC6908934

[9] Wong K van der Weyden L Schott CR Foote A Constantino-Casas F Smith S. Cross-species genomic landscape comparison of human mucosal melanoma with canine oral and equine melanoma. Nat Commun. (2019) 10:353. 10.1038/s41467-018-08081-1 30664638 PMC6341101

[10] Hernandez B Adissu HA Wei BR Michael HT Merlino G Simpson RM. Naturally occurring canine melanoma as a predictive comparative oncology model for human mucosal and other triple wild-type melanomas. Int J Mol Sci. (2018) 19:20394. 10.3390/ijms19020394 29385676 PMC5855616

[11] Mikiewicz M Pazdzior-Czapula K Gesek M Lemishevskyi V Otrocka-Domagala I. Canine and feline oral cavity tumours and tumour-like lesions: a retrospective study of 486 cases (2015–2017). J Comp Pathol. (2019) 172:80–7. 10.1016/j.jcpa.2019.09.007 31690420

[12] Spangler WL Kass PH. The histologic and epidemiologic bases for prognostic considerations in canine melanocytic neoplasia. Vet Pathol. (2006) 43:136–49. 10.1354/vp.43-2-136 16537931

[13] Turek M LaDue T Looper J Nagata K Shiomitsu K Keyerleber M. Multimodality treatment including ONCEPT for canine oral melanoma: a retrospective analysis of 131 dogs. Vet Radiol Ultrasound. (2020) 61:471–80. 10.1111/vru.12860 32323424

[14] Proulx DR Ruslander DM Dodge RK Hauck ML Williams LE Horn B. A retrospective analysis of 140 dogs with oral melanoma treated with external beam radiation. Vet Radiol Ultrasound. (2003) 44:352–9. 10.1111/j.1740-8261.2003.tb00468.x 12816381

[15] Bergman PJ. Cancer immunotherapies. Vet Clin North Am Small Anim Pract. (2019) 49:881–902. 10.1016/j.cvsm.2019.04.010 31186125

[16] Grosenbaugh DA Leard AT Bergman PJ Klein MK Meleo K Susaneck S. Safety and efficacy of a xenogeneic DNA vaccine encoding for human tyrosinase as adjunctive treatment for oral malignant melanoma in dogs following surgical excision of the primary tumor. Am J Vet Res. (2011) 72:1631–8. 10.2460/ajvr.72.12.1631 22126691

[17] Tsao H Atkins MB Sober AJ. Management of cutaneous melanoma. N Engl J Med. (2004) 351:998–1012. 10.1056/NEJMra041245 15342808

[18] Park JS Withers SS Modiano JF Kent MS Chen M Luna JI. Canine cancer immunotherapy studies: linking mouse and human. J Immunother Cancer. (2016) 4:97. 10.1186/s40425-016-0200-7 28031824 PMC5171656

[19] Moore PF Olivry T Naydan D. Canine cutaneous epitheliotropic lymphoma (mycosis fungoides) is a proliferative disorder of CD8+ T cells. Am J Pathol. (1994) 144:421–9. 7906096 PMC1887150

[20] McSweeney PA Rouleau KA Wallace PM Bruno B Andrews RG Krizanac-Bengez L. Characterization of monoclonal antibodies that recognize canine CD34. Blood. (1998) 91:1977–86. 10.1182/blood.V91.6.1977 9490680

[21] Cobbold S Metcalfe S. Monoclonal antibodies that define canine homologues of human CD antigens: summary of the First International Canine Leukocyte Antigen Workshop (CLAW). Tissue Antigens. (1994) 43:137–54. 10.1111/j.1399-0039.1994.tb02315.x 8091414

[22] Wilkerson MJ Dolce K Koopman T Shuman W Chun R Garrett L. Lineage differentiation of canine lymphoma/leukemias and aberrant expression of CD molecules. Vet Immunol Immunopathol. (2005) 106:179–96. 10.1016/j.vetimm.2005.02.020 15963817

[23] Vernau W Moore PF. An immunophenotypic study of canine leukemias and preliminary assessment of clonality by polymerase chain reaction. Vet Immunol Immunopathol. (1999) 69:145–64. 10.1016/S0165-2427(99)00051-3 10507302

[24] Moore PF Rossitto PV Danilenko DM Wielenga JJ Raff RF Severns E. Monoclonal antibodies specific for canine CD4 and CD8 define functional T-lymphocyte subsets and high-density expression of CD4 by canine neutrophils. Tissue Antigens. (1992) 40:75–85. 10.1111/j.1399-0039.1992.tb01963.x 1412420

[25] Choi JW Withers SS Chang H Spanier JA De La Trinidad VL Panesar H. Development of canine PD-1/PD-L1 specific monoclonal antibodies and amplification of canine T cell function. PLoS ONE. (2020) 15:e0235518. 10.1371/journal.pone.0235518 32614928 PMC7332054

[26] Foltz JA Somanchi SS Yang Y Aquino-Lopez A Bishop EE Lee DA. NCR1 expression identifies canine natural killer cell subsets with phenotypic similarity to human natural killer cells. Front Immunol. (2016) 7:521. 10.3389/fimmu.2016.00521 27933061 PMC5120128

[27] Igase M Nemoto Y Itamoto K Tani K Nakaichi M Sakurai M. A pilot clinical study of the therapeutic antibody against canine PD-1 for advanced spontaneous cancers in dogs. Sci Rep. (2020) 10:18311. 10.1038/s41598-020-75533-4 33110170 PMC7591904

[28] Maekawa N Konnai S Nishimura M Kagawa Y Takagi S Hosoya K. PD-L1 immunohistochemistry for canine cancers and clinical benefit of anti-PD-L1 antibody in dogs with pulmonary metastatic oral malignant melanoma. NPJ Precis Oncol. (2021) 5:10. 10.1038/s41698-021-00147-6 33580183 PMC7881100

[29] Goulart MR Hlavaty SI Chang YM Polton G Stell A Perry J. Phenotypic and transcriptomic characterization of canine myeloid-derived suppressor cells. Sci Rep. (2019) 9:3574. 10.1038/s41598-019-40285-3 30837603 PMC6400936

[30] Grondahl-Rosado C Boysen P Johansen GM Brun-Hansen H Storset AK. NCR1 is an activating receptor expressed on a subset of canine NK cells. Vet Immunol Immunopathol. (2016) 177:7–15. 10.1016/j.vetimm.2016.05.001 27436439

[31] Ito D Brewer S Modiano JF Beall MJ. Development of a novel anti-canine CD20 monoclonal antibody with diagnostic and therapeutic potential. Leuk Lymphoma. (2015) 56:219–25. 10.3109/10428194.2014.914193 24724777 PMC5002357

[32] Rue SM Eckelman BP Efe JA Bloink K Deveraux QL Lowery D. Identification of a candidate therapeutic antibody for treatment of canine B-cell lymphoma. Vet Immunol Immunopathol. (2015) 164:148–59. 10.1016/j.vetimm.2015.02.004 25764941

[33] Withers SS Moore PF Chang H Choi JW McSorley SJ Kent MS. Multi-color flow cytometry for evaluating age-related changes in memory lymphocyte subsets in dogs. Dev Comp Immunol. (2018) 87:64–74. 10.1016/j.dci.2018.05.022 29859828 PMC6197816

[34] Pantelyushin S Ranninger E Bettschart-Wolfensberger R Vom Berg J. OMIP-065: dog immunophenotyping and T-cell activity evaluation with a 14-color panel. Cytometry A. (2020) 97:1024–7. 10.1002/cyto.a.24168 32583607

[35] Wu Y Chang YM Stell AJ Priestnall SL Sharma E Goulart MR. Phenotypic characterisation of regulatory T cells in dogs reveals signature transcripts conserved in humans and mice. Sci Rep. (2019) 9:13478. 10.1038/s41598-019-50065-8 31530890 PMC6748983

[36] Avery PR Burton J Bromberek JL Seelig DM Elmslie R Correa S. Flow cytometric characterization and clinical outcome of CD4+ T-cell lymphoma in dogs: 67 cases. J Vet Intern Med. (2014) 28:538–46. 10.1111/jvim.12304 24495161 PMC4857986

[37] Haran KP Lockhart A Xiong A Radaelli E Savickas PJ Posey A. Generation and validation of an antibody to canine CD19 for diagnostic and future therapeutic purposes. Vet Pathol. (2020) 57:241–52. 10.1177/0300985819900352 32081102 PMC7462180

[38] Durgeau A Virk Y Corgnac S Mami-Chouaib F. Recent advances in targeting CD8 T-cell immunity for more effective cancer immunotherapy. Front Immunol. (2018) 9:14. 10.3389/fimmu.2018.00014 29403496 PMC5786548

[39] Xia A Zhang Y Xu J Yin T Lu XJT. Cell dysfunction in cancer immunity and immunotherapy. Front Immunol. (2019) 10:1719. 10.3389/fimmu.2019.01719 31379886 PMC6659036

[40] Waldman AD Fritz JM Lenardo MJ. A guide to cancer immunotherapy: from T cell basic science to clinical practice. Nat Rev Immunol. (2020) 20:651–68. 10.1038/s41577-020-0306-5 32433532 PMC7238960

[41] Dow S. A role for dogs in advancing cancer immunotherapy research. Front Immunol. (2019) 10:2935. 10.3389/fimmu.2019.02935 32010120 PMC6979257

[42] Monjazeb AM Kent MS Grossenbacher SK Mall C Zamora AE Mirsoian A. Blocking indolamine-2,3-dioxygenase rebound immune suppression boosts antitumor effects of radio-immunotherapy in murine models and spontaneous canine malignancies. Clin Cancer Res. (2016) 22:4328–40. 10.1158/1078-0432.CCR-15-3026 26979392 PMC5010514

[43] Wickham H. Ggplot2: Elegant Graphics for Data Analysis, Use R!. Cham: Springer (2016). p. 1. 10.1007/978-3-319-24277-4_9

[44] Rabiger FV Rothe K von Buttlar H Bismarck D Buttner M Moore PF. Distinct features of canine non-conventional CD4(-)CD8alpha(-) double-negative TCRalphabeta(+) vs. TCRgammadelta(+) T cells. Front Immunol. (2019) 10:2748. 10.3389/fimmu.2019.02748 31824515 PMC6883510

[45] Hertoghs KM Moerland PD van Stijn A Remmerswaal EB Yong SL van de Berg PJ. Molecular profiling of cytomegalovirus-induced human CD8+ T cell differentiation. J Clin Invest. (2010) 120:4077–90. 10.1172/JCI42758 20921622 PMC2964975

[46] Hurkmans DP Basak EA Schepers N Oomen-De Hoop E Van der Leest CE El Bouazzaoui S. Granzyme B is correlated with clinical outcome after PD-1 blockade in patients with stage IV non-small-cell lung cancer. J Immunother Cancer. (2020) 8:586. 10.1136/jitc-2020-000586 32461348 PMC7254154

[47] Iga N Otsuka A Yamamoto Y Nakashima C Honda T Kitoh A. Accumulation of exhausted CD8+ T cells in extramammary Paget's disease. PLoS ONE. (2019) 14:e0211135. 10.1371/journal.pone.0211135 30682105 PMC6347258

[48] Prizment AE Vierkant RA Smyrk TC Tillmans LS Nelson HH Lynch CF. Cytotoxic T cells and granzyme B associated with improved colorectal cancer survival in a prospective cohort of older women. Cancer Epidemiol Biomarkers Prev. (2017) 26:622–31. 10.1158/1055-9965.EPI-16-0641 27979806 PMC5380516

[49] Miller BC Sen DR Al Abosy R Bi K Virkud YV LaFleur MW. Subsets of exhausted CD8(+) T cells differentially mediate tumor control and respond to checkpoint blockade. Nat Immunol. (2019) 20:326–36. 10.1038/s41590-019-0312-6 30778252 PMC6673650

[50] Tamang DL Redelman D Alves BN Vollger L Bethley C Hudig D. Induction of granzyme B and T cell cytotoxic capacity by IL-2 or IL-15 without antigens: multiclonal responses that are extremely lytic if triggered and short-lived after cytokine withdrawal. Cytokine. (2006) 36:148–59. 10.1016/j.cyto.2006.11.008 17188506 PMC1850105

[51] Masuda K Yasuda N. The antibody against human CD25, ACT-1, recognizes canine T-lymphocytes in the G2/M and G0/G1 phases of the cell cycle during proliferation. J Vet Med Sci. (2008) 70:1285–7. 10.1292/jvms.70.1285 19057154

[52] Mizuno T Suzuki R Umeki S Okuda M. Crossreactivity of antibodies to canine CD25 and Foxp3 and identification of canine CD4+CD25 +Foxp3+ cells in canine peripheral blood. J Vet Med Sci. (2009) 71:1561–8. 10.1292/jvms.001561 20046022

[53] Rissetto KC Rindt H Selting KA Villamil JA Henry CJ Reinero CR. Cloning and expression of canine CD25 for validation of an anti-human CD25 antibody to compare T regulatory lymphocytes in healthy dogs and dogs with osteosarcoma. Vet Immunol Immunopathol. (2010) 135:137–45. 10.1016/j.vetimm.2010.02.002 20197202

[54] Abrams VK Hwang B Lesnikova M Gass MJ Wayner E Castilla-Llorente C. Novel monoclonal antibody specific for canine CD25 (P4A10): selection and evaluation of canine Tregs. Vet Immunol Immunopathol. (2010) 135:257–65. 10.1016/j.vetimm.2009.12.006 20060595 PMC2864801

[55] Jeurink PV Vissers YM Rappard B Savelkoul HFT. cell responses in fresh and cryopreserved peripheral blood mononuclear cells: kinetics of cell viability, cellular subsets, proliferation, and cytokine production. Cryobiology. (2008) 57:91–103. 10.1016/j.cryobiol.2008.06.002 18593572

[56] Sadeghi A Ullenhag G Wagenius G Totterman TH Eriksson F. Rapid expansion of T cells: effects of culture and cryopreservation and importance of short-term cell recovery. Acta Oncol. (2013) 52:978–86. 10.3109/0284186X.2012.737020 23126547

[57] Biller BJ Elmslie RE Burnett RC Avery AC Dow SW. Use of FoxP3 expression to identify regulatory T cells in healthy dogs and dogs with cancer. Vet Immunol Immunopathol. (2007) 116:69–78. 10.1016/j.vetimm.2006.12.002 17224188

[58] Horiuchi Y Tominaga M Ichikawa M Yamashita M Okano K Jikumaru Y. Relationship between regulatory and type 1 T cells in dogs with oral malignant melanoma. Microbiol Immunol. (2010) 54:152–9. 10.1111/j.1348-0421.2009.00194.x 20236425

[59] Milevoj N Tratar UL Nemec A Brozic A Znidar K Sersa G. combination of electrochemotherapy, gene electrotransfer of plasmid encoding canine IL-12 and cytoreductive surgery in the treatment of canine oral malignant melanoma. Res Vet Sci. (2019) 122:40–9. 10.1016/j.rvsc.2018.11.001 30453179

[60] Tominaga M Horiuchi Y Ichikawa M Yamashita M Okano K Jikumaru Y. Flow cytometric analysis of peripheral blood and tumor-infiltrating regulatory T cells in dogs with oral malignant melanoma. J Vet Diagn Invest. (2010) 22:438–41. 10.1177/104063871002200317 20453222

[61] Yasumaru CC Xavier JG Strefezzi RF Salles-Gomes COM. Intratumoral T-lymphocyte subsets in canine oral melanoma and their association with clinical and histopathological parameters. Vet Pathol. (2021) 58:491–502. 10.1177/0300985821999321 33764216

[62] Porcellato I Silvestri S Menchetti L Recupero F Mechelli L Sforna M. Tumour-infiltrating lymphocytes in canine melanocytic tumours: an investigation on the prognostic role of CD3(+) and CD20(+) lymphocytic populations. Vet Comp Oncol. (2020) 18:370–80. 10.1111/vco.12556 31750993

[63] Sakai K Maeda S Yamada Y Chambers JK Uchida K Nakayama H. Association of tumour-infiltrating regulatory T cells with adverse outcomes in dogs with malignant tumours. Vet Comp Oncol. (2018) 16:330–6. 10.1111/vco.12383 29322606

[64] Maibach F Sadozai H Seyed Jafari SM Hunger RE Schenk M. Tumor-infiltrating lymphocytes and their prognostic value in cutaneous melanoma. Front Immunol. (2020) 11:2105. 10.3389/fimmu.2020.02105 33013886 PMC7511547

[65] Paluskievicz CM Cao X Abdi R Zheng P Liu Y Bromberg JS. Regulatory cells and priming the suppressive tumor microenvironment. Front Immunol. (2019) 10:2453. 10.3389/fimmu.2019.02453 31681327 PMC6803384

[66] Li X Zheng Y. Regulatory T cell identity: formation and maintenance. Trends Immunol. (2015) 36:344–53. 10.1016/j.it.2015.04.006 25981968 PMC4458194

[67] Togashi Y Shitara K Nishikawa H. Regulatory T cells in cancer immunosuppression – implications for anticancer therapy. Nat Rev Clin Oncol. (2019) 16:356–71. 10.1038/s41571-019-0175-7 30705439

[68] Gregg R Smith CM Clark FJ Dunnion D Khan N Chakraverty R. The number of human peripheral blood CD4+ CD25high regulatory T cells increases with age. Clin Exp Immunol. (2005) 140:540–6. 10.1111/j.1365-2249.2005.02798.x 15932517 PMC1809384

[69] Reddy M Eirikis E Davis C Davis HM Prabhakar U. Comparative analysis of lymphocyte activation marker expression and cytokine secretion profile in stimulated human peripheral blood mononuclear cell cultures: an in vitro model to monitor cellular immune function. J Immunol Methods. (2004) 293:127–42. 10.1016/j.jim.2004.07.006 15541283

[70] Rothe K Bismarck D Buttner M Alber G von Buttlar H. Canine peripheral blood CD4(+)CD8(+) double-positive Tcell subpopulations exhibit distinct Tcell phenotypes and effector functions. Vet Immunol Immunopathol. (2017) 185:48–56. 10.1016/j.vetimm.2017.01.005 28242002

[71] Tarpataki N Wawrzyniak M Akdis CA Ruckert B Meli ML Fischer NM. The effects of cryopreservation on the expression of canine regulatory T-cell markers. Vet Dermatol. (2017) 28:396–e93. 10.1111/vde.12438 28317209

[72] Bajnok A Ivanova M Rigo J Toldi G. The distribution of activation markers and selectins on peripheral T lymphocytes in preeclampsia. Mediators Inflamm. (2017) 2017:8045161. 10.1155/2017/8045161 28555090 PMC5438859

[73] Obar JJ Molloy MJ Jellison ER Stoklasek TA Zhang W Usherwood EJ. CD4+ T cell regulation of CD25 expression controls development of short-lived effector CD8+ T cells in primary and secondary responses. Proc Natl Acad Sci USA. (2010) 107:193–8. 10.1073/pnas.0909945107 19966302 PMC2806751

[74] Shatrova AN Mityushova EV Vassilieva IO Aksenov ND Zenin VV Nikolsky NN. Time-dependent regulation of IL-2R alpha-chain (CD25) expression by TCR signal strength and IL-2-induced STAT5 signaling in activated human blood T lymphocytes. PLoS ONE. (2016) 11:e0167215. 10.1371/journal.pone.0167215 27936140 PMC5172478

[75] Snook JP Kim C Williams MA. TCR signal strength controls the differentiation of CD4(+) effector and memory T cells. Sci Immunol. (2018) 3:aas9103. 10.1126/sciimmunol.aas9103 30030369 PMC6126666

[76] Triplett TA Curti BD Bonafede PR Miller WL Walker EB Weinberg AD. Defining a functionally distinct subset of human memory CD4+ T cells that are CD25POS and FOXP3NEG. Eur J Immunol. (2012) 42:1893–905. 10.1002/eji.201242444 22585674

[77] Castro F Cardoso AP Goncalves RM Serre K Oliveira MJ. Interferon-gamma at the crossroads of tumor immune surveillance or evasion. Front Immunol. (2018) 9:847. 10.3389/fimmu.2018.00847 29780381 PMC5945880

[78] Attridge K Walker LS. Homeostasis and function of regulatory T cells (Tregs) *in vivo*: lessons from TCR-transgenic Tregs. Immunol Rev. (2014) 259:23–39. 10.1111/imr.12165 24712457 PMC4237543

[79] Vukmanovic-Stejic M Agius E Booth N Dunne PJ Lacy KE Reed JR. The kinetics of CD4+Foxp3+ T cell accumulation during a human cutaneous antigen-specific memory response *in vivo*. J Clin Invest. (2008) 118:3639–50. 10.1172/JCI35834 18924611 PMC2556297

[80] Santegoets SJ Dijkgraaf EM Battaglia A Beckhove P Britten CM Gallimore A. Monitoring regulatory T cells in clinical samples: consensus on an essential marker set and gating strategy for regulatory T cell analysis by flow cytometry. Cancer Immunol Immunother. (2015) 64:1271–86. 10.1007/s00262-015-1729-x 26122357 PMC4554737

[81] Lanier LL Chang C Spits H Phillips JH. Expression of cytoplasmic CD3 epsilon proteins in activated human adult natural killer (NK) cells and CD3 gamma, delta, epsilon complexes in fetal NK cells. Implications for the relationship of NK and T lymphocytes. J Immunol. (1992) 149:1876–80. 1387664

[82] De Smedt M Taghon T Van de Walle I De Smet G Leclercq G Plum J. Notch signaling induces cytoplasmic CD3 epsilon expression in human differentiating NK cells. Blood. (2007) 110:2696–703. 10.1182/blood-2007-03-082206 17630354

[83] Morice WG. The immunophenotypic attributes of NK cells and NK-cell lineage lymphoproliferative disorders. Am J Clin Pathol. (2007) 127:881–6. 10.1309/Q49CRJ030L22MHLF 17509985

[84] Shin DJ Park JY Jang YY Lee JJ Lee YK Shin MG. Ex vivo expansion of canine cytotoxic large granular lymphocytes exhibiting characteristics of natural killer cells. Vet Immunol Immunopathol. (2013) 153:249–59. 10.1016/j.vetimm.2013.03.006 23548866 PMC3769186

[85] Huang YC Hung SW Jan TR Liao KW Cheng CH Wang YS. CD5-low expression lymphocytes in canine peripheral blood show characteristics of natural killer cells. J Leukoc Biol. (2008) 84:1501–10. 10.1189/jlb.0408255 18708592

[86] Lee SH Shin DJ Kim Y Kim CJ Lee JJ Yoon MS. Comparison of phenotypic and functional characteristics between canine non-B, non-T natural killer lymphocytes and CD3(+)CD5(dim)CD21(-) cytotoxic large granular lymphocytes. Front Immunol. (2018) 9:841. 10.3389/fimmu.2018.00841 29755462 PMC5934500

[87] Canter RJ Grossenbacher SK Foltz JA Sturgill IR Park JS Luna JI. Radiotherapy enhances natural killer cell cytotoxicity and localization in pre-clinical canine sarcomas and first-in-dog clinical trial. J Immunother Cancer. (2017) 5:98. 10.1186/s40425-017-0305-7 29254507 PMC5735903

[88] Kryczek I Liu R Wang G Wu K Shu X Szeliga W. FOXP3 defines regulatory T cells in human tumor and autoimmune disease. Cancer Res. (2009) 69:3995–4000. 10.1158/0008-5472.CAN-08-3804 19383912

[89] Bettini M Vignali DA. Regulatory T cells and inhibitory cytokines in autoimmunity. Curr Opin Immunol. (2009) 21:612–8. 10.1016/j.coi.2009.09.011 19854631 PMC2787714

[90] Okeke EB Uzonna JE. The pivotal role of regulatory T cells in the regulation of innate immune cells. Front Immunol. (2019) 10:680. 10.3389/fimmu.2019.00680 31024539 PMC6465517

[91] Nemoto Y Shosu K Okuda M Noguchi S Mizuno T. Development and characterization of monoclonal antibodies against canine PD-1 and PD-L1. Vet Immunol Immunopathol. (2018) 198:19–25. 10.1016/j.vetimm.2018.02.007 29571514

[92] Coy J Caldwell A Chow L Guth A Dow S. PD-1 expression by canine T cells and functional effects of PD-1 blockade. Vet Comp Oncol. (2017) 15:1487–502. 10.1111/vco.12294 28120417

