# Supplementary material for: T Cell Immune Profiles of Blood and Tumor in Dogs Diagnosed With Malignant Melanoma
Source: Front Vet Sci. 2021 Dec 2;8:772932. doi: 10.3389/fvets.2021.772932 (PMC8674490; doi:10.3389/fvets.2021.772932)
Supplement: Supplementary file 1 [file Data_Sheet_1.PDF]

## Supplementary Figure 1

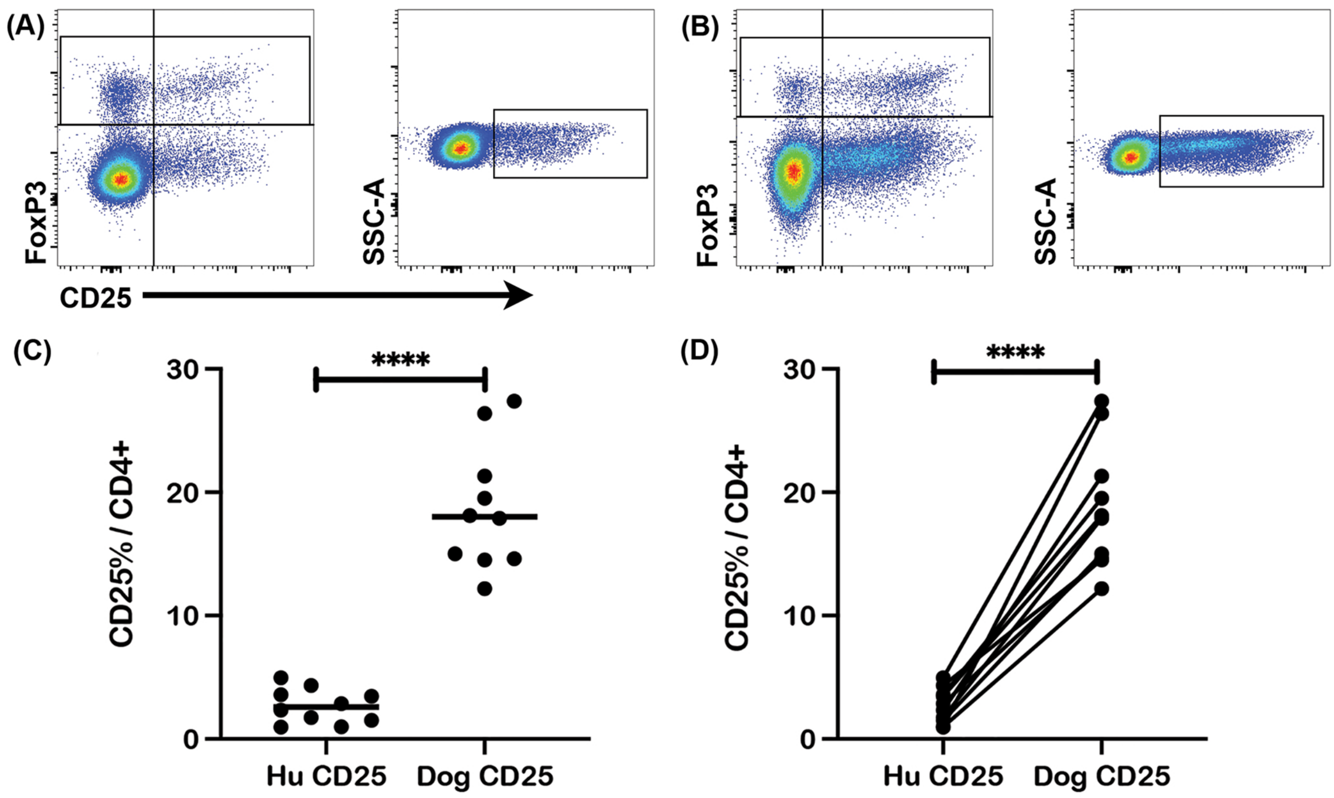


**Supplementary Figure 1.** Comparison staining of canine CD4+ T cell subsets in blood with an anti-canine CD25 and anti-human CD25 monoclonal antibodies. Healthy donor PBMC were isolated for flow cytometry and stained for viability, CD3, CD4 CD8, CD25 (either anti-canine or anti-human) and FoxP3. CD4+ cells gated from a parental CD3+ population were next interrogated for FoxP3+ and CD25+ populations. Representative scatter plots demonstrate interrogation of gated canine CD4+ T cells for FoxP3 and CD25 (left-hand panel), or for CD25 alone against side scatter area-area (SSC-A) (right-hand panel) using either (A) anti-human CD25 monoclonal antibody (clone ACT1) or (B) anti-canine CD25 monoclonal antibody (clone P4A10) for a single healthy dog. (C) Median frequency of CD25+ cells within the CD4+ T cell subset based on staining with the anti-human CD25 antibody (Hu CD25) is compared with frequency based on staining with anti-canine CD25 antibody (Dog CD25) in PBMC from healthy control dogs (n = 10). (D) CD25+ cell frequency for CD4+ T cells determined by Hu CD25 antibody are matched to frequency determined by Dog CD25 antibody for each healthy control. Pair-wise analysis between frequencies based on Hu CD25 and Dog CD25 antibodies was conducted with the Mann Whitney test using GraphPad Prism software and two-tailed analysis. Four asterisks (****) denote a P value $< 0.0001$. P values $< 0.05$ are considered significant.

## Supplementary Figure 2

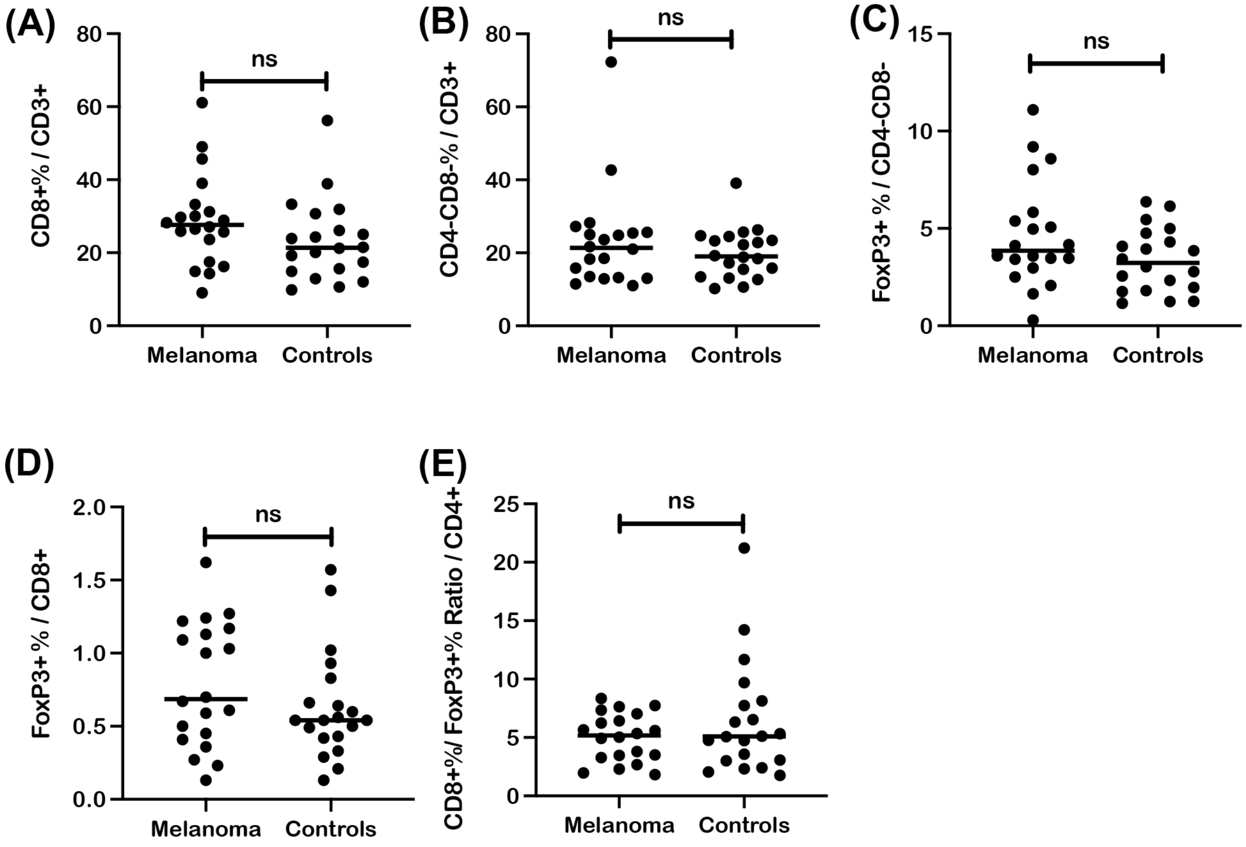


**Supplementary Figure 2.** Frequencies of T cell subsets including regulatory subsets in blood from canine melanoma patients and healthy controls. A comparison of median frequencies for (A) CD8+ cells and (B) CD4-CD8- cells within the T cell population for patients and controls is shown. Median frequencies of FoxP3+ cells within (C) CD4-CD8- and (D) CD8+ T cell populations are also compared between patients and healthy controls. (E) Ratios of CD8+ frequency in T cells to frequency of FoxP3+ cells in CD4+ T cells are shown with median values described for patients and healthy controls. Pair-wise analysis between patients and controls was conducted with the Mann Whitney test using GraphPad Prism software and two-tailed analysis. "ns" denotes not significant.

## Supplementary Figure 3

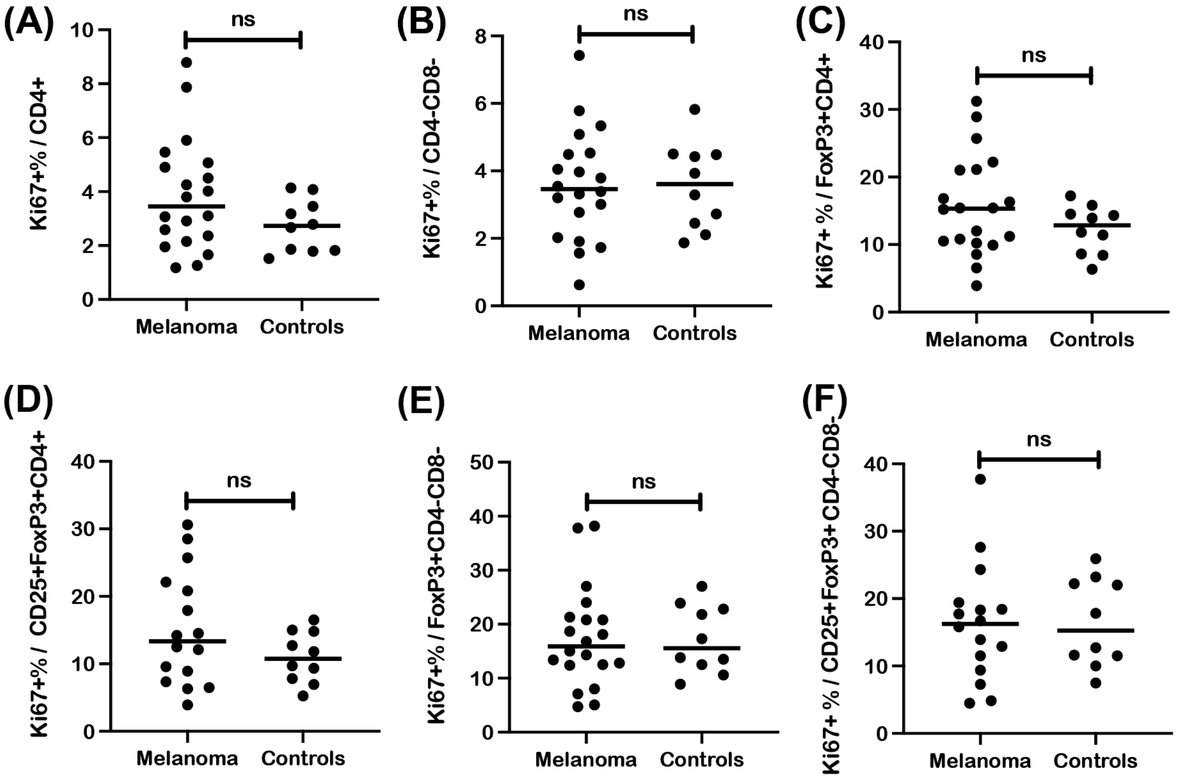


**Supplementary Figure 3.** Frequencies of Ki67 expressing cells within regulatory T cell subsets in blood from canine melanoma patients and healthy controls. Median frequencies of Ki67+ cells within (A) CD4+ and (B) CD4-CD8- T cell subsets are compared between patients and healthy controls. Median frequencies of Ki67+ cells within (C) Foxp3+CD4+, (D) CD25+FoxP3+CD4+, (E) FoxP3+CD4-CD8-, and (F) CD25+FoxP3+CD4-CD8- T cells are also compared between patients and healthy controls. Pair-wise analysis between patients and controls was conducted with the Mann Whitney test using GraphPad Prism software and two-tailed analysis. "ns" denotes not significant.

**Supplementary Figure 4**

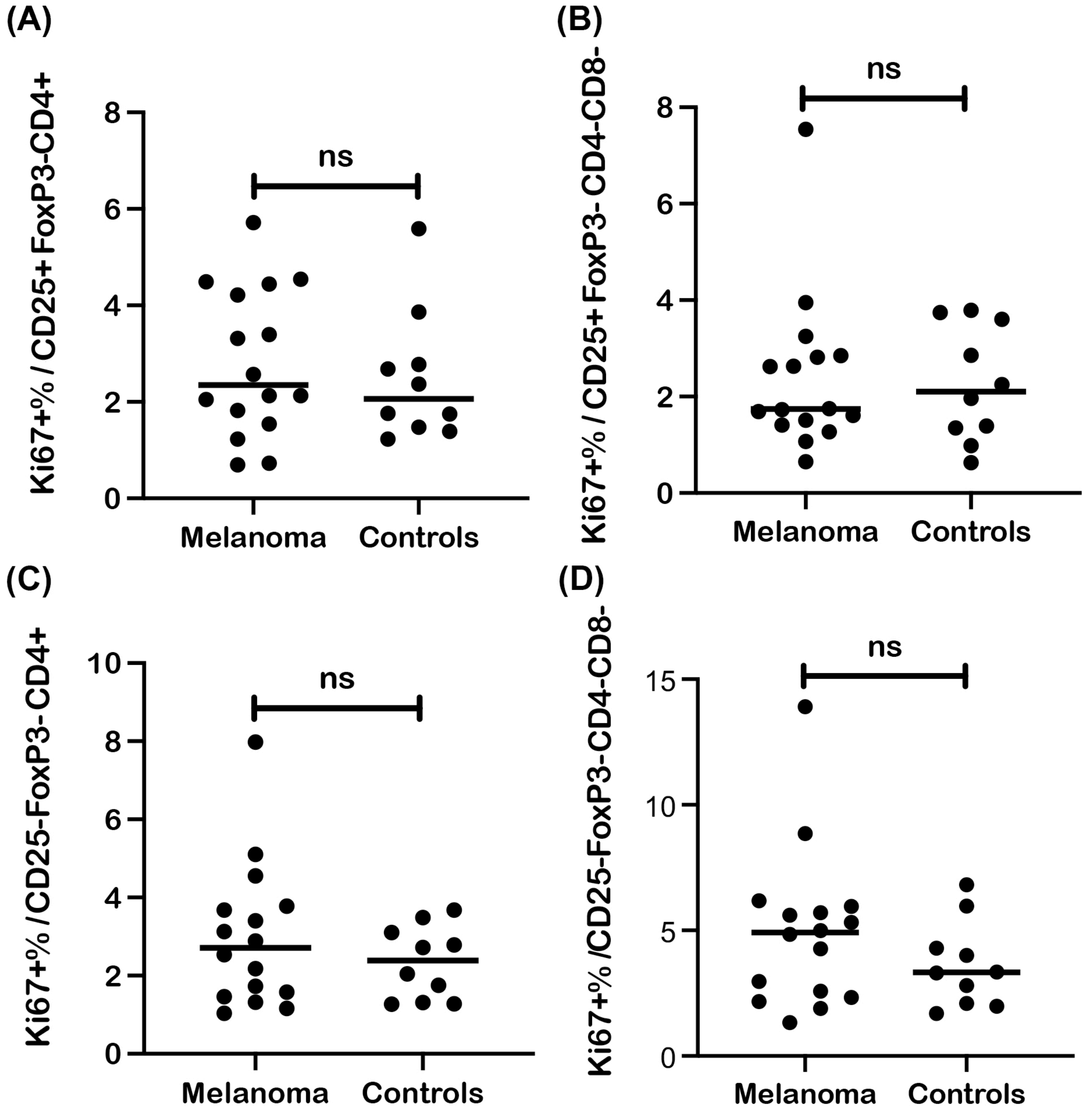


**Supplementary Figure 4.** Frequencies of Ki67 expressing cells within activated and quiescent T cell subsets in blood from canine melanoma patients and healthy controls. Median frequencies of Ki67+ cells within (A) CD25+FoxP3-CD4+ and (B) CD25+FoxP3-CD4-CD8- activated T cell subsets are compared between patients and healthy controls. Median frequencies of Ki67+ cells within (C) CD25-FoxP3-CD4+ and (D) CD25-FoxP3-CD4-CD8-quiescent T cell subsets are also compared between patients and healthy controls. Pair-wise analysis between patients and controls was conducted with the Mann Whitney test using GraphPad Prism software and two-tailed analysis. "ns" denotes not significant.

## Supplementary Figure 5

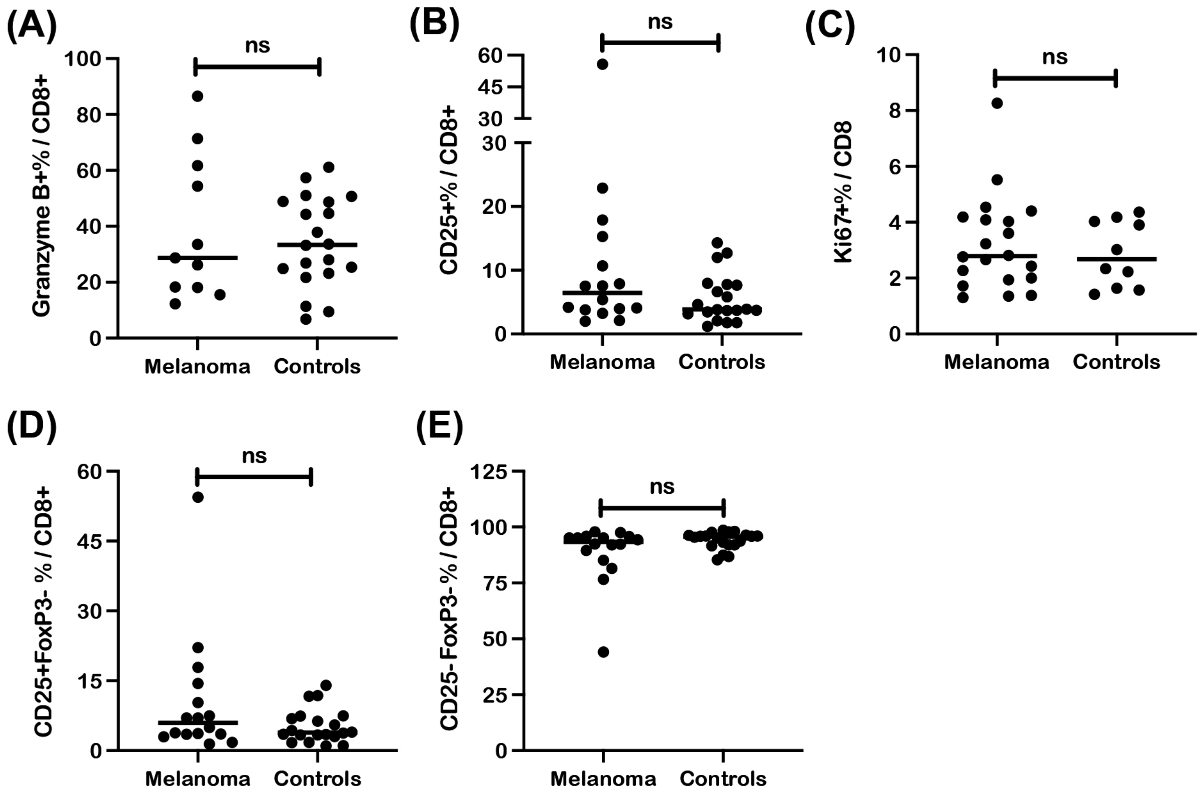


**Supplementary Figure 5.** Frequencies of activated and quiescent CD8+ T cell subsets in blood from canine melanoma patients and healthy controls. Median frequencies of (A) granzyme B+, (B) CD25+, and (C) Ki67+ within the CD8+ T cell subset are compared between patients and healthy controls. Median frequencies of (D) CD25+FoxP3- and (E) CD25-FoxP3- cells within the CD8+ T cell subset are compared between patients and healthy controls. Pair-wise analysis between patients and controls was conducted with the Mann Whitney test using GraphPad Prism software and two-tailed analysis. "ns" denotes not significant.

## Supplementary Figure 6

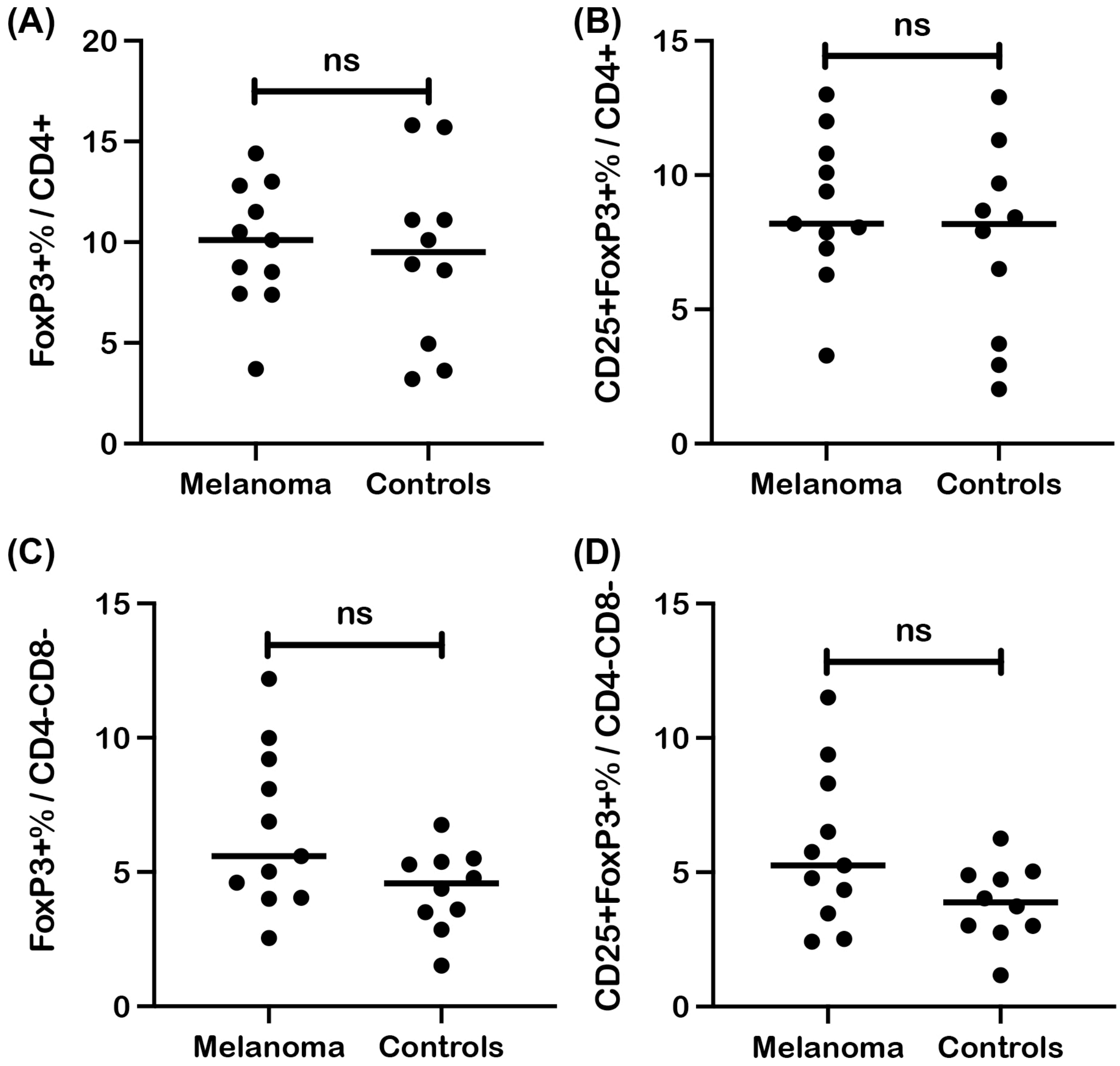


**Supplementary Figure 6.** Frequencies of regulatory T cell subsets after ConA stimulation in blood from canine melanoma patients and healthy controls. Median frequencies of (A) FoxP3+ and (B) CD25+FoxP3+ cells after ConA stimulation were determined for CD4+ T cells and compared between patients and healthy controls. Median frequencies of (C) FoxP3+ and (D) CD25+FoxP3+ cells after ConA stimulation were also determined for the CD4-CD8- T cell subset and compared between patients and healthy controls. Pair-wise analysis between patients and controls was conducted with the Mann Whitney test using GraphPad Prism software and two-tailed analysis. "ns" denotes not significant.

Supplementary Figure 7

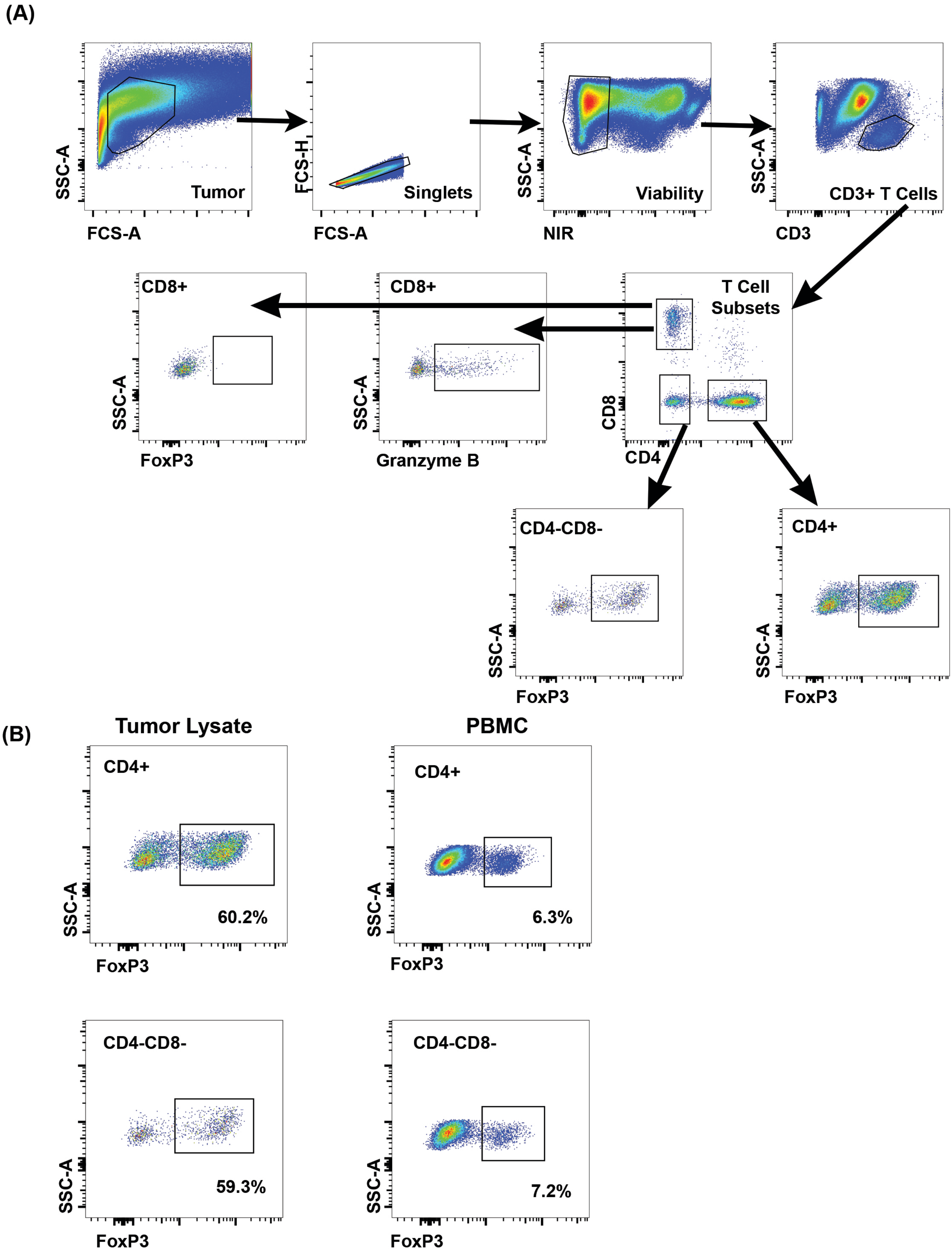


**Supplementary Figure 7.** Gating strategies for interrogation of canine T cell subsets by flow cytometry in tumor lysate and blood. Flow cytometric analysis of fresh tumor lysate prepared from a melanoma patient biopsy and PBMC isolated from the same patient are shown. (A) Representative scatter plots reveal the gating strategy for detection of CD4+, CD8+, and CD4-CD8- T cell subsets starting with establishment of a tumor gate, followed by gating on singlets, exclusion of dead cells by a viability stain and gating on CD3+ cells as the parental gate for T cell subsets. Scatter plots further show interrogation of all T cell subsets for FoxP3 and CD8+ T cells for granzyme-B. (B) Scatter plots representing FoxP3+ cell populations within the CD4+ and CD4-CD8- T cell subsets derived from tumor lysate and PBMC from the same canine melanoma patient are shown.

## Supplementary Figure 8

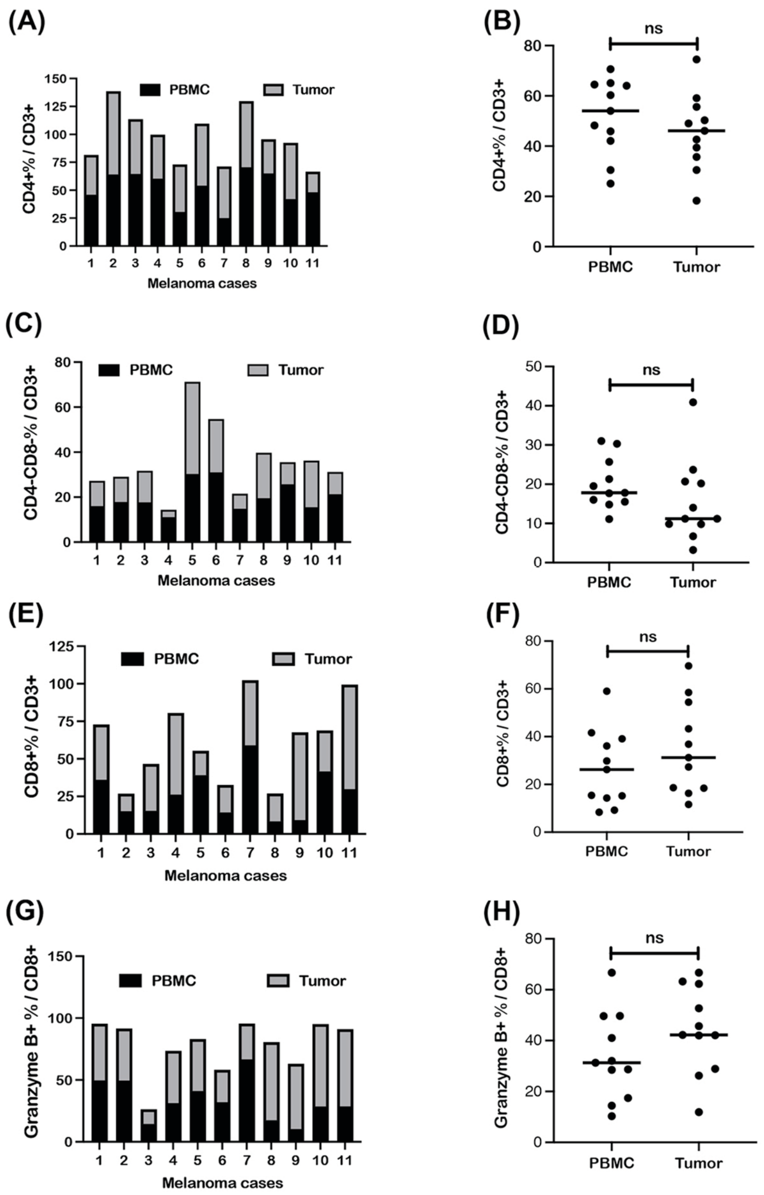


**Supplementary Figure 8.** Comparison of frequencies of T cell subsets in tumor lysate and blood in canine melanoma patients. PBMC isolated from blood and tumor lysates from a separate cohort of 11 canine melanoma patients were assessed for T cell phenotypes by flow cytometry. (A) A stacked bar analysis of frequencies of CD4+ cells within the T cell population in blood versus tumor lysate from each patient is shown for all patients. (B) Median frequencies of CD4+ cells within the T cell population are compared between PBMC and tumor as a grouped analysis for the same patients. (C) A stacked bar analysis of frequencies of CD4-CD8- cells within the T cell population in blood versus tumor lysate from each patient is shown for all patients. (D) Median frequencies of CD4-CD8- cells within the T cell population are compared between PBMC and tumor as a grouped analysis for the same patients. (E) A stacked bar analysis of frequencies of CD8+ cells within the T cell population in blood versus tumor lysate from each patient is shown for all patients. (F) Median frequencies of CD8+ cells within the T cell population are compared between PBMC and tumor as a grouped analysis for the same patients. (G) A stacked bar analysis of frequencies of granzyme B+ cells within the CD8+ T cell subset in blood versus tumor lysate from each patient is shown for all patients. (H) Median frequencies of granzyme B+ cells within the CD8+ T cell subset are compared between PBMC and tumor as a grouped analysis for the same patients. Pair-wise analysis between frequencies for PBMC and tumor lysate was conducted with the Mann Whitney test using GraphPad Prism software and two-tailed analysis. "ns" denotes not significant.

## Supplementary Figure 9

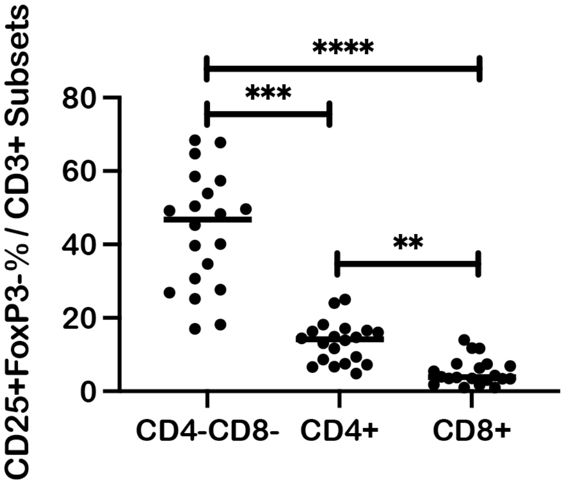


**Supplementary Figure 9.** Association between CD25 expression and specific T cell subsets in healthy dog PBMC. CD4-CD8-, CD4+, and CD8+ T cells subsets are compared for median frequencies of CD25+FoxP3- cells. Comparisons of frequencies between subsets were performed with a Kruskal-Wallis test (one-way ANOVA) with Dunn's post hoc test for multiple comparisons using GraphPad Prism software. Two asterisks (**) denote a P value < 0.01; three asterisks (***) denote a P value < 0.001 and four asterisks (****) denote a P value < 0.0001. P values < 0.05 are considered significant.

## Supplementary Table 1

**Supplementary Table 1.** Demographic information on patients and control dogs and tumor information on patient dogs. – indicates information was not available. NA indicates not applicable. For the sex column F indicates an intact female, FS indicates an spayed female and MC indicates a castrated male dog. An X in the metastasis at diagnosis column indicates metastatic disease was identified at the time of diagnosis for all dogs but one this was lymph node metastasis.

| Patients (P) and Controls (C) Designation | Age (Years) | Breed | Sex | Weight (kg) | Tumor Location | Largest Tumor Measurement (cm) | Mitotic Index (Mitosis/10 HPF) | Metastasis at diagnosis |
|---|---|---|---|---|---|---|---|---|
| **P1** | 13.6 | Cocker Spaniel | FS | 12.5 | Oral | 3 | - | X |
| **P2** | 8.3 | Golden Retriever | MC | 41.0 | Dermal | 1 | 3 | |
| **P3** | 10.0 | Mixed | MC | 24.7 | Oral | 1 | - | |
| **P4** | 11.3 | Yorkshire Terrier | MC | 7.8 | Oral | 1.5 | 3 | |
| **P5** | 9.8 | Samoyed | FS | 21.2 | Oral | 1 | - | |
| **P6** | 12.8 | Pomeranian | FS | 4.9 | Oral | 3 | 9 | |
| **P7** | 15.1 | Mixed | FS | 16.5 | Anal gland | 2 | 4.8 | |
| **P8** | 9.7 | Miniature Schnauzer | FS | 6.4 | Digit | 0.5 | 2.5 | |
| **P9** | 9.4 | Labrador Retriever | MC | 40.2 | Oral | 5 | 2 | X |
| **P10** | 8.7 | Boxer | FS | 29.5 | Unknown Primary | 0 | - | X |
| **P11** | 13.5 | Mixed | FS | 20.1 | Oral | 1.5 | 28 | X |
| **P12** | 10.1 | English Cocker Spaniel | FS | 10.7 | Oral | 7 | 3 | X |
| **P13** | 10.2 | Mixed | FS | 7.5 | Oral | 1.5 | - | X |
| **P14** | 10.4 | Irish Terrier | MI | 16 | Oral | 0.8 | 0 | |
| **P15** | 8.7 | Mixed | MC | 30 | Oral | 1.9 | 1 | |
| **P16** | 7.1 | Mixed | MC | 50 | Oral | 4 | - | |
| **P17** | 5.9 | Mixed | F | 6.2 | Oral | 3.5 | 20 | |
| **P18** | 12.5 | English Cocker Spaniel | MC | 11.7 | Oral | 3.5 | 5 | |
| **P19** | 10.6 | Basset Hound | FS | 25.4 | Oral | 1.5 | 8 | |
| **P20** | 13.5 | Soft Coated Wheaten Terrier | MC | 20.9 | Dermal | 0.7 | 20 | |
| **P21** | 8.0 | Australian Shepherd | FS | 27.3 | Oral | 2.5 | 40 | |
| **P22** | 9.0 | Labrador Retriever | FS | 43.1 | Oral | 3 | 37 | X |
| **P23** | 12.0 | Golden Retriever | MC | 37.2 | Oral | 2.5 | 0 | |
| **P24** | 9.5 | French Bulldog | MC | 10 | Oral | - | - | |
| **P25** | 12.4 | Labrador Retriever | FS | 34.4 | Oral | 5 | - | |
| **P26** | 9.3 | Mixed | MC | 33.3 | Oral | 4 | - | |
| **P27** | 6.8 | Boxer | MC | 30.7 | Oral | 5.5 | 10 | X |
| **P28** | 12.2 | Weimaraner | MC | 23 | Oral | 4 | 21 | |
| **P29** | 12.1 | Mixed | MC | 32.6 | Oral | 5.3 | 9 | X |
| **P30** | 11.0 | Mixed | MC | 26.3 | Oral | 3.5 | 20 | X |
| **P31** | 13.3 | Labrador Retriever | FS | 30.3 | Oral | 5 | 3 | |
| **C1** | 7 | English Setter | FS | - | NA | NA | NA | NA |
| **C2** | 6.5 | Doberman Pinscher | MC | 38 | NA | NA | NA | NA |
| **C3** | 6 | Bloodhound | FS | 27.3 | NA | NA | NA | NA |
| **C4** | 4.8 | Australian Shepherd | FS | - | NA | NA | NA | NA |
| **C5** | 4 | Mixed | FS | - | NA | NA | NA | NA |
| **C6** | 9.5 | Mixed | FS | 5.2 | NA | NA | NA | NA |
| **C7** | 8 | French Bulldog | MC | 10 | NA | NA | NA | NA |
| **C8** | 6.5 | Beagle | FS | 11.2 | NA | NA | NA | NA |
| **C9** | 11 | Border Terrier | MC | 8.5 | NA | NA | NA | NA |
| **C10** | 9.3 | Mixed | MC | 29.5 | NA | NA | NA | NA |
| **C11** | 9.8 | Rat Terrier | MC | 14.9 | NA | NA | NA | NA |
| **C12** | 8.3 | Golden Retriever | MC | 47 | NA | NA | NA | NA |
| **C13** | 15.5 | Mixed | MC | 11.1 | NA | NA | NA | NA |
| **C14** | 8.5 | Mixed | MC | 18.6 | NA | NA | NA | NA |
| **C15** | 14.8 | Golden Retriever | FS | - | NA | NA | NA | NA |
| **C16** | 13 | Golden Retriever | MC | 30 | NA | NA | NA | NA |
| **C17** | 10.1 | Briard | FS | 28.5 | NA | NA | NA | NA |
| **C18** | 14 | Standard Poodle | FS | 12.5 | NA | NA | NA | NA |
| **C19** | 10 | Mixed | FS | 31.5 | NA | NA | NA | NA |
| **C20** | 8 | Australian Shepherd | MC | 21.7 | NA | NA | NA | NA |
| **C21** | 9.5 | Rat Terrier | FS | 7.2 | NA | NA | NA | NA |
| **C22** | 7.8 | Dachshund Mix | MC | 6.3 | NA | NA | NA | NA |
| **C23** | 5.7 | Rat Terrier | MC | 8.5 | NA | NA | NA | NA |

## Supplementary Table 2:

**Supplementary Table 2:** Patients and controls included in analysis. PCA Resting indicates cases included in the principal component analysis (PCA) using resting cells and PCA Stim indicates cases used to analyze ConA stimulated cells. – indicates that there was not enough sample to use a particular antibody or that a sample was not available. NA designates that a sample was not applicable.

| Patients (P) and Controls (C) Designation | CD3 | CD4 | CD8 | FoxP3 | KI67 | CD25 (dog) | CD25 (human) | Granzyme B | Interferon-γ | Blood | Tumor | PCA No ICS Panel | PCA ICS Panel |
|---|---|---|---|---|---|---|---|---|---|---|---|---|---|
| **P1** | X | X | X | X | - | - | X | - | - | X | - | | |
| **P2** | X | X | X | X | - | - | X | - | - | X | - | | |
| **P3** | X | X | X | X | - | - | X | - | - | X | - | | |
| **P4** | X | X | X | X | - | - | X | - | - | X | - | | |
| **P5** | X | X | X | X | X | X | NA | - | - | X | - | X | |
| **P6** | X | X | X | X | X | X | NA | - | - | X | - | X | |
| **P7** | X | X | X | X | X | X | NA | - | - | X | - | X | |
| **P8** | X | X | X | X | X | X | NA | - | - | X | - | X | |
| **P9** | X | X | X | X | X | X | NA | - | - | X | - | X | |
| **P10** | X | X | X | X | X | X | NA | X | X | X | - | X | X |
| **P11** | X | X | X | X | X | X | NA | X | X | X | - | X | X |
| **P12** | X | X | X | X | X | X | NA | X | X | X | - | X | X |
| **P13** | X | X | X | X | X | X | NA | X | X | X | - | X | X |
| **P14** | X | X | X | X | X | X | NA | X | X | X | - | X | X |
| **P15** | X | X | X | X | X | X | NA | X | X | X | - | X | X |
| **P16** | X | X | X | X | X | X | NA | X | X | X | - | X | X |
| **P17** | X | X | X | X | X | X | NA | X | X | X | - | X | X |
| **P18** | X | X | X | X | X | X | NA | X | X | X | - | X | X |
| **P19** | X | X | X | X | X | X | NA | X | X | X | - | X | X |
| **P20** | X | X | X | X | X | X | NA | X | X | X | - | X | X |
| **P21** | X | X | X | X | - | - | NA | X | - | X | X | | |
| **P22** | X | X | X | X | - | - | NA | X | - | X | X | | |
| **P23** | X | X | X | X | - | - | NA | X | - | X | X | | |
| **P24** | X | X | X | X | - | - | NA | X | - | X | X | | |
| **P25** | X | X | X | X | - | - | NA | X | - | X | X | | |
| **P26** | X | X | X | X | - | - | NA | X | - | X | X | | |
| **P27** | X | X | X | X | - | - | NA | X | - | X | X | | |
| **P28** | X | X | X | X | - | - | NA | X | - | X | X | | |
| **P29** | X | X | X | X | - | - | NA | X | - | X | X | | |
| **P30** | X | X | X | X | - | - | NA | X | - | X | X | | |
| **P31** | X | X | X | X | - | - | NA | X | - | X | X | | |
| **C1** | X | X | X | X | X | X | X | X | X | X | NA | X | X |
| **C2** | X | X | X | X | X | X | X | X | X | X | NA | X | X |
| **C3** | X | X | X | X | X | X | X | X | X | X | NA | X | X |
| **C4** | X | X | X | X | X | X | X | X | X | X | NA | X | X |
| **C5** | X | X | X | X | X | X | X | X | X | X | NA | X | X |
| **C6** | X | X | X | X | X | X | X | X | X | X | NA | X | X |
| **C7** | X | X | X | X | X | X | X | X | X | X | NA | X | X |
| **C8** | X | X | X | X | X | X | X | - | - | X | NA | X | |
| **C9** | X | X | X | X | X | X | X | - | - | X | NA | X | |
| **C10** | X | X | X | X | X | X | X | - | - | X | NA | X | |
| **C11** | X | X | X | X | X | X | NA | X | - | X | NA | X | |
| **C12** | X | X | X | X | X | X | NA | X | - | X | NA | X | |
| **C13** | X | X | X | X | X | X | NA | X | - | X | NA | X | |
| **C14** | X | X | X | X | X | X | NA | X | - | X | NA | X | |
| **C15** | X | X | X | X | X | X | NA | X | - | X | NA | X | |
| **C16** | X | X | X | X | X | X | NA | X | - | X | NA | X | |
| **C17** | X | X | X | X | X | X | NA | X | - | X | NA | X | |
| **C18** | X | X | X | X | X | X | NA | X | - | X | NA | X | |
| **C19** | X | X | X | X | X | X | NA | X | - | X | NA | X | |
| **C20** | X | X | X | X | X | X | NA | X | - | X | NA | X | |
| **C21** | X | X | X | X | X | X | NA | X | X | X | NA | X | X |
| **C22** | X | X | X | X | X | X | NA | X | X | X | NA | X | X |
| **C23** | X | X | X | X | X | X | NA | X | X | X | NA | X | X |

## Supplementary Table 3

**Supplementary Table 3.** Flow cytometry parameters included in the principal component analysis (PCA) shown in Figure 2. – indicates that when background was subtracted the value was <0 indicating no stimulation.

| Parameters in PCA Analysis | Figure Panels A & C | Figure Panel B | Figure Panel D |
|---|---|---|---|
| CD4+% / CD3+ | x | x | x |
| FoxP3+% / CD4+ | x | x | x |
| CD4-CD8-% / CD3+ | x | x | x |
| FoxP3+% / CD4-CD8- | x | x | x |
| CD8+% / CD3+ | x | x | x |
| FoxP3+% / CD8+ | x | x | x |
| CD25+% / CD4+ | x | x | x |
| FoxP3+CD25+% /CD4+ | x | x | x |
| CD25+FoxP3-% / CD4+ | x | x | x |
| CD25-FoxP3-% / CD4+ | x | - | - |
| CD25+% / CD4-CD8- | x | x | x |
| FoxP3+CD25+% / CD4-CD8- | x | x | x |
| FoxP3-CD25+% / CD4-CD8- | x | x | x |
| FoxP3-CD25-% / CD4-CD8- | x | x | x |
| CD25+% / CD8+ | x | x | x |
| FoxP3-CD25+% / CD8+ | x | x | x |
| FoxP3-CD25-% / CD8+ | x | x | - |
| Granzyme-B+% / CD4-CD8- | | x | x |
| Granzyme-B+% / CD8+ | | x | x |
| IFN-γ+% / CD4+ | | x | x |
| IFN-γ+% / FoxP3-CD25+CD4+ | | x | x |
| IFN-γ+% / CD4-CD8- | | x | x |
| IFN-γ+% / FoxP3-CD25+CD4-CD8- | | x | x |
| IFN-γ+% / CD8+ | | x | x |
